# Honey Volatiles as a Fingerprint for Botanical Origin—A Review on their Occurrence on Monofloral Honeys

**DOI:** 10.3390/molecules25020374

**Published:** 2020-01-16

**Authors:** Alexandra M. Machado, Maria Graça Miguel, Miguel Vilas-Boas, Ana Cristina Figueiredo

**Affiliations:** 1 Centro de Estudos do Ambiente e do Mar (CESAM Lisboa), Faculdade de Ciências da Universidade de Lisboa, Centro de Biotecnologia Vegetal (CBV), DBV, C2, Piso 1, Campo Grande, 1749-016 Lisboa, Portugal; ialexam@gmail.com; 2 Faculdade de Ciências e Tecnologia, Mediterranean Institute for Agriculture, Environment and Development, Universidade do Algarve, Campus de Gambelas, 8005-139 Faro, Portugal; mgmiguel@ualg.pt; 3 CIMO, Centro de Investigação de Montanha, Instituto Politécnico de Bragança, Campus de Santa Apolónia, 5300-253 Bragança, Portugal; mvboas@ipb.pt

**Keywords:** honey volatiles, monofloral honey, botanical source, marker compounds, honey authenticity

## Abstract

Honeys have specific organoleptic characteristics, with nutritional and health benefits, being highly appreciated by consumers, not only in food but also in the pharmaceutical and cosmetic industries. Honey composition varies between regions according to the surrounding flora, enabling its characterization by source or type. Monofloral honeys may reach higher market values than multifloral ones. Honey’s aroma is very specific, resulting from the combination of volatile compounds present in low concentrations. The authentication of honey’s complex matrix, according to its botanical and/or geographical origin, represents a challenge nowadays, due to the different sorts of adulteration that may occur, leading to the search for reliable marker compounds for the different monofloral honeys. The existing information on the volatiles of monofloral honeys is scarce and disperse. In this review, twenty monofloral honeys and honeydews, from acacia, buckwheat, chestnut, clover, cotton, dandelion, eucalyptus, fir tree, heather, lavender, lime tree, orange, pine, rape, raspberry, rhododendron, rosemary, strawberry tree, sunflower and thyme, were selected for volatile comparison purposes. Taking into consideration the country of origin, the technique of isolation and analysis, the five main volatiles from each of the honeys are compared. Whereas some compounds were found in several types of monofloral honey, and thus not considered good volatile markers, some monofloral honeys revealed characteristic volatile compounds independently of their provenance.

## 1. Introduction

Honey is defined as “the natural sweet substance produced by *Apis mellifera* bees from the nectar of plants or from secretions of living parts of plants or excretions of plant-sucking insects on the living parts of plants, which the bees collect, transform by combining with specific substances of their own, deposit, dehydrate, store and leave in honeycombs to ripen and mature”, according to the Council Directive 2001/110/EC relating to honey produced and marketed in the European Union (EU) [1]. Both the EU [1] and the Codex Alimentarius Commission [2] set compositional criteria for honey, which basically comprises a concentrated water solution of two main sugars, fructose and glucose, with small amounts of various complex sugars, as well as other constituents such as enzymes, amino acids, organic acids, carotenoids, vitamins, minerals, volatile compounds, pollen and wax [3,4,5,6,7,8,9]. Honey has been reported to also contain a variety of flavonoids and phenolic acids that exhibit a wide range of biological properties and are responsible for its antioxidant and anti-inflammatory properties [10,11,12,13,14].

Honey composition is strongly associated with its botanical source and the geographical area of collection. Chemical volatile composition has great importance in characterizing honey’s botanical source [15,16,17], which directly influences its organoleptic characteristics [18,19,20,21].

According to the geographical origin of production, honey can be linked with specific areas within the EU, under the labels of Protected Designation of Origin (PDO) and Protected Geographical Identification (PGI). Honeys stamped with these labels generally present characteristics that are essentially or exclusively related to a specific region or a particular local environment with inherent natural and human factors [22]. At present, Portugal is the country with the highest number of honeys registered in the EU (nine PDO honeys), followed by Spain (five PDO and one PGI honeys) and France (two PDO and three PGI honeys) [22].

Honey from bees which collect most of the nectar from a certain type of flower is called monofloral and possesses distinctive organoleptic characteristics, like highly distinguishing aromas, probably derived from the nectar, indicating the presence of volatile components responsible for their characteristic fragrances, and thus is considered as a premium product [7,23,24,25]. Contrarily, multifloral honey is obtained when bees collect nectar from different types of flowers. In addition, we may also find honey from sugar exudates, so called honeydew or forest honey, which is normally collected from insect’s sweet exudates. However, the most widely available honey in the market is blended honey, consisting of a homogenous mixture of two or more honeys with different characteristics such as geographical origin, botanical source, colour, flavour or density [25,26].

The consumer demand for monofloral honey has increased in recent years, due to its particular flavour and pharmacological properties, increasing its commercial value [11,27]. This fact may induce adulterations with low-cost and nutritionally valuable substances or mislabelling concerning the botanical origin [28]. To overcome this fraud, the scientific community has strengthened the research on the development of reliable methodologies and chemical markers that may contribute for honey discrimination, indicating floral and/or geographic origin [29,30]. These would allow the obtaining of a standard of quality and authenticity for honey, protecting the consumer from fraudulent mislabelling of inferior honey, trying to raise its market value [21,31,32].

The International Honey Commission (IHC) is encouraging the development of harmonized analytical methods of quality certification for different honeys. The assessment of honey’sbotanical origin is of great importance in food analysis, particularly in honey, since authenticity guarantees the quality [33].

In order to evaluate the organoleptic quality and authenticity of a food product, the aroma profile is one of the most typical characteristics; the volatile compounds are the main responsible for this organoleptic feature, contributing to flavour, along with taste and physical factors [25,34]. Honey’s aroma is one important factor for its differentiation as a function of botanical origin.

Research on honey volatiles began in the early 1960s and it was found that volatiles could originate from a number of factors, such as: a) nectar or honeydew collected by honeybees and linked to the plant characteristics, b) the transformation of plant compounds by the honeybee or directly generated by honeybee, including during honey processing or storage, or c) from microbial or environmental contamination [25,33,35]. Therefore, the volatile compound profile can potentially be used as a fingerprint for honey authentication, which could enable the identification of a honey’s origin [11,21,36,37].

The present review details the key factors that influence honey volatiles, the isolation and analytical procedures, and compares the volatile constituents of specific monofloral honeys from different countries. Aiming at trying to unravel the importance of botanical source, geographical origin and isolation/analysis techniques in monofloral honey volatiles characterization, the selected twenty monofloral honeys, Table 1, were chosen considering a minimum requirement of two different countries of provenance, for comparison purposes on marker volatile compounds.

## 2. Factors that Influence Honey Volatiles

Different research works have shown that it is difficult to establish reliable volatile compounds as chemical markers for honeys obtained from several botanical sources [38,39]. This is mainly due to the chemical composition of honey being dependent not only on the botanical source, but also on geographical origin, harvesting season, storage conditions, possible interactions between chemical compounds in the honey that occur naturally and also during thermal processing [30,40]. Furthermore, compounds belonging to different chemical classes are dependent on a honeybee’s metabolism but also on technical processing, which includes volatile extraction and analysis [41].

Moreover, different volatile isolation procedures and analysis techniques may lead to different results, obtaining more than one class of marker compounds in different proportions for honeys with the same botanical origin [23,35,42]. The importance of these factors on honey volatiles is discussed in the three following sections.

### 2.1. Botanical Source—Honey’s Characteristic Aroma Profiles

The chemical composition of honey is highly dependent on the floral origin of the nectar foraged by bees. However, honey is often marketed as mixed-flower honey with a blend of flavours. In order to determine the legitimacy of the botanical source of honey, analyses of pollen (melissopalynology) and organoleptic or physicochemical properties are traditionally employed [43]. Melissopalynology requires a very experienced analyst, is very time consuming, and depends on the expert’s ability and judgment. Honey melissopalynological analysis, based on the identification and quantification of the pollen percentage by microscopic examination, is a very useful method to determine the geographical origin of honeys [44]. However, pollen analysis may not give enough information for several honey types obtained from plant genus with underrepresented or overrepresented pollen, such as *Citrus* spp., *Robinia pseudoacacia*, *Arbutus unedo* or *Castanea sativa* [45,46,47,48]. Consequently, there is a need for detailed chemical characterization of these honeys, and the identification of volatile compounds can provide an additional tool in its authentication [49,50,51].

Owing to the high number of volatile components in honey, the aroma profile may represent a fingerprint of the product, which could be used to determine its origin [9,52]. Several authors have argued that the careful analysis of honey´s volatile compounds could be a useful tool for characterization of botanical origin [53]. This determination based on the aroma profile is particularly dependable for a flavour-rich product such as honey and has led to the development of techniques for measuring its volatile fraction [52].

### 2.2. Honey Volatiles Not Derived from Bee Processing or from Honey Botanical Source

Several factors may contribute to the presence of volatile compounds in honey, which are not directly related to the bee processing or to the honey botanical source, namely (a) thermal processing, (b) storage conditions (temperature and storage period), and (c) extraction techniques. During thermal processing or prolonged storage, labile compounds may be oxidized or destroyed, and volatile compounds could be produced by Maillard reactions and by Strecker degradation reactions [20,54].

Volatiles extraction and analysis techniques may also contribute to the formation of certain compounds not related with the honey botanical source. Heating during an hydrodistillation, might induce the formation of artefacts, mainly due to the thermal degradation of sugars [55].

Carbohydrates and free amino acids are responsible for the generation of furan and pyran derivatives, mostly hydroxymethylfurfural (HMF), whilst Strecker degradation reactions occurring between amino acids and dicarbonyl compounds can produce aliphatic and aromatic aldehydes [20,30,56]. The use of extraction methods that apply heat during this procedure as in hydrodistillation (HD) or microsimultaneous steam distillation–solvent extraction (MSDE), could also contribute to the formation of these furan and pyran derivatives [55,56].

### 2.3. Isolation and Analysis of Honey Volatiles

The isolation of volatile compounds from a complex matrix like honey is very difficult and so different methods can be used, with different degrees of selectivity and efficacy [35,57]. Humid-heat-based extraction procedures, such as hydrodistillation (HD) or distillation–extraction [58,59] are common techniques for volatile isolation. Other methods, such as dynamic headspace extraction (DHE) and headspace solid-phase microextraction (HS-SPME), perform a partitioning of volatiles between honey and the above vapour phase [35,59]. Ultrasound-assisted extraction (USE) is a methodology also mentioned in the literature, which significantly reduces extraction times and improves it, when compared with traditional methods, due to the mechanical effect of the ultrasound on the extraction solvent, enhancing the penetration of the solvent into the matrix via cavitation [60].

Most studies herewith reviewed used HS-SPME to isolate the volatile fraction, because it has some advantages over other methods, such as its simplicity, being free of organic solvents, allowing for the quantification of a large number of molecules with little or no handling of samples whilst significantly decreasing the extraction time [20,61]. Other studies used different extraction techniques [HD, liquid–liquid extraction (LLE), solid-phase extraction (SPE), USE], that provide complementary information concerning honey volatiles, as they are based on diverse principles [60,62,63,64].

The techniques most used to identify and quantify isolated honey volatiles compounds, following the extraction procedure, are gas chromatography (GC) and gas chromatography-coupled mass spectrometry (GC-MS). In this review, the data is compared based on the frequency of occurrence of specific compounds for each monofloral honey, since a direct comparison based on the quantification of the identified volatiles was not possible: some studies performed quantification by GC or GC-MS, others reported the data in percentages or in absolute amounts and, in other cases, no quantification was made.

## 3. Main Volatile Compounds in Monofloral Honeys from Different Geographical Origins

Aroma compounds are present in honey at very low concentrations and are seen as complex mixtures of volatile components of different functionalities and relatively low molecular weights [57]. Among these are compounds from distinct biosynthetic pathways, such as terpenoids and phenolic derivatives, obtained via mevalonic and shikimic acid pathways, respectively, with several functional groups, such as alcohols, aldehydes, ketones, esters, carboxylic acids, benzene derivatives and nitrogen containing compounds [65]. Currently, more than 600 volatile compounds have been identified in honeys from different botanical origins [66]. In recent years, several works have been published describing the volatile profiles of different monofloral honeys, which highlights the growing interest in this subject [50,52,53,54,57]. As stated in Section 2.1, the percentage of pollen grains needed to classify a honey as monofloral is variable, ranging from 8% to 20% of *Arbutus* pollen for strawberry tree honey, >45% of *Erica* pollen for heather honey and >86% of *Castanea* pollen for chestnut honey [44]. Given the new challenges in the international market or domestic regulations, which may demand a higher percentage of dominant pollen grains for the characterization of particular monofloral honey types, volatile compound analysis may provide a faster and more accurate tool for botanical and geographical origin identification, by identifying the specific volatile metabolites [67].

In this section, the characteristic aroma profiles of honeys from twenty different botanical sources are reviewed (Table 1). Different isolation procedures and quantification methods were used to describe each honey´s volatiles in the different studies. Therefore, herein the comparison will be performed in terms of the frequency of occurrence of specific compounds in each monofloral honey from different countries of provenance, in order to summarize the most important volatile components that characterize them (Tables 2–21). Therefore, each table corresponds to a monofloral honey or honeydew type and is organised according to the country of origin of the samples as well the botanical source (in some cases discriminating honey from honeydew), number of samples studied, melissopalynological examination, volatile isolation procedure and corresponding analysis. Finally, the five dominant volatile compounds identified for each sample are described (the five main quantitative compounds independently of the measure units used by the authors-percentage, μg/100 g, μg/kg or other units), which do not always correspond to the marker compounds of the analysed honey types. Although in Tables 2–21 only the five dominant compounds are presented for each sample belonging to a specific monofloral honey, other compounds that are not among the dominant ones, but which are common to honeys from different countries, are also mentioned.

### 3.1. Acacia Honey

Honey from acacia, characterized by a sweet, beeswax and sourish flavour [41], produced in thirteen countries from three continents, namely Europe, Africa and Asia, was studied, aiming at the identification of characteristic volatile compounds (Table 2). The oxygen-containing monoterpene *cis*-linalool oxide, the alcohol 3-methyl-3-buten-1-ol and the aldehyde heptanal, detected in acacia honey samples from twelve of the thirteen countries [52,68,69,70,71,72,73], were considered to be marker volatile compounds. *cis*-Linalool oxide was identified in honeys from Austria, Czech Republic, France, Germany, Italy, Morocco, Poland, Romania, Slovakia and Spain. 3-methyl-3-buten-1-ol was isolated in honeys from Austria, Czech Republic, Romania and Spain. Heptanal was detected in honeys from France, Germany, Hungary, Italy, Poland, Slovakia and Romania. Unlike most of the samples, acacia honey from China [74] did not present any of these three volatile compounds.

Benzaldehyde, furfural, hexanal, octanal, nonanal, decanal were compounds frequently reported in this type of honey. Ethanol was also identified in several honeys, which could indicate a fermentation process [75].

### 3.2. Buckwheat Honey

Buckwheat honey is characterized by a distinctive flavour, often defined as pungent, sweetish and malty [71,80], which is attributed to some specific volatile compounds. Aldehydes like 3-methylbutanal, 2-methylbutanal [72,77,80,81,82] and the short-chain carboxylic acid isovaleric acid (3-methylbutyric acid) [77,80,81] were the most common constituents in buckwheat honeys analysed by the different studies from four countries (Table 3).

### 3.3. Chestnut Honey

Chestnut honey is characterized by a bitter, sweet, burnt caramel and woody flavour [52,83]. The main volatiles present in this honey (Table 4) include benzaldehyde, which was identified in all the samples from the eight countries, 2-aminoacetophenone was present in honey samples from Croatia, Italy, Portugal, Spain and France [21,40,78,79,83,84], and acetophenone in Croatia, France, Germany, Italy and Spain samples [21,41,52,75,78,79,83,85,86]. 1-phenylethanol was also identified in chestnut honeys from Croatia, France, Germany, Italy, Spain and Turkey [41,52,60,75,79,83,85,87], phenylacetic acid in honeys from Croatia, France, Italy and Spain [21,60,85,86], 3-hexen-1-ol was detected in honeys from France, Germany, Italy and Spain [52,79] and 2-methyldihydrofuranone in honeys produced in France, Germany and Italy [52]. These compounds were the most frequently reported in this type of honey, indicating that they may be considered volatile marker compounds of chestnut honey.

### 3.4. Clover Honey

*Trifolium repens* (white clover) and *Trifolium pratense* (red clover) are two species of the same genus; however, different volatiles were identified in honeys obtained from each species as the main botanical source. According to the literature (Table 5), white clover honey is characterized by an abundance in benzene derivatives, namely methoxybenzaldehyde, benzyl alcohol [89], phenylacetaldehyde, benzaldehyde [55,76], methyl benzoate, methyl 2-methoxybenzoate, benzoic acid and 2-hydroxy-3-phenylpropionic acid [90]. Red clover honey presents as its main volatile compounds lilac aldehyde isomers, followed by phenylacetaldehyde and benzaldehyde [91].

### 3.5. Cotton Honey

The literature has reported only four studies concerning cotton honey’s volatile compounds, from Greece, Palestine and Spain (Table 6) [87,88,92,93]. This honey has a mild aroma and very sweet taste [93]. Despite the small number of studies, compounds like nonanal, phenylacetaldehyde and phenylethyl alcohol were common to all samples from the three countries.

### 3.6. Dandelion honey

The main volatile constituents of dandelion honey, which has a mild aroma and a very sweet taste [41] (Table 7), include nitriles in higher amounts, namely 3-methylpentanenitrile [79,94] and phenylacetonitrile [75,94] and, in lower amounts, methyl branched aliphatic acids like 2-methylbutanoic acid, 2-methylpropanoic acid, and 3-methylbutanoic acid [41,79,94]. It is nevertheless important to mention that the only study that did not report these volatile compounds [76] used DHS, whereas all the others used SPME.

### 3.7. Eucalyptus Honey

Eucalyptus honey, with an intense and moderately persistent flavour, tasting of soft caramel and mushrooms [83], shows several classes of volatile compounds [62,78,79,84,95,96], some of them common between honeys from different countries (Table 8). Among these were aldehydes (nonanal in samples from Italy, Spain, Palestine and Turkey, decanal in samples from Italy, Portugal and Palestine, phenylacetaldehyde in those from Australia, Palestine, Spain and Italy), alcohols and carboxylic acids (nonanol and nonanoic acid respectively, both in Italy, Portugal and Spain samples, while in samples from Palestine only nonanoic acid was identified), terpenes (*p*-cymene and borneol, both in samples from Italy and Spain, linalool and linalool oxides in those from Australia, Italy, Morocco, Portugal and Spain) and norisoprenoids (α-isophorone and 4-oxoisophorone in Italy, Portugal and Spain samples, 3-oxo-α-ionone in samples from Australia and Spain, 3-oxo-α-ionol in those from Australia, Italy and Spain). High amounts of diketones and hydroxyketones were also identified in eucalyptus honeys, such as 3-hydroxy-2-butanone (acetoin) (Australia, Italy, Spain), 3-hydroxy-5-methyl-2-hexanone and 2-hydroxy-5-methyl-3-hexanone (Spain and Italy) [97].

### 3.8. Fir Tree Honey and Honeydew

Sixteen fir tree honey and honeydew samples, provided by local beekeepers from five countries, were analysed by means of GC-MS (Table 9). Of these samples, only those from Croatia and France reported melissopalynological analyses. This honey is characterized by a sweet, caramel, slightly bitter and a little sourish flavour [41].

According to the qualitative data analysis, numerous volatile compounds were identified in the different studies (Table 9), such as aldehydes (isobutanal, heptanal), alcohols (3-methylbutanol, 2-ethyl-1-hexanol, 2-ethyl-1-hexanol), benzene derivatives (benzene acetaldehyde, salicylaldehyde), carboxylic acids (acetic acid, 2-methylbutanoic acid), hydrocarbons (hexane, octane), esters (nonanoic acid ethyl ester), ketones (2,3-butanedione), furan derivatives (dihydro-2(3H)-furanone, furfural), terpenes (*trans*-linalool oxide, α-pinene), norisoprenoids (4-oxoisophorone). Nevertheless, no common marker compounds were identified in the honeys from the five countries. Furan-related compounds could be a result of honey processing like heat treatment on sugar or amino acids, characteristic of non-enzymatic browning reactions, and also due to storage [20,68,101,102].

### 3.9. Heather Honey

Nectar from the genera *Erica* and *Calluna* contribute to the production of heather honey [100], its flavour being characterized by sweet and candy-like notes [105]. The identified volatile compounds in this honey comprise several ones also present in other types of monofloral honey, including carboxylic acids (2-methylpropanoic acid, phenylacetic acid, 2-hydroxy-3-phenylpropionic acid, butyric acid phenylacetic acid, decanoic acid) and benzene derivatives (benzaldehyde, benzyl alcohol, benzoic acid, benzeneacetic acid) (Table 10).

Norisoprenoids, such as α-isophorone, 4-oxoisophorone, 4-hydroxy-4-(3-oxo-1-butenyl)-3,5,5-trimethylcyclohex-2-en-1-one, 4-(3-oxo-1-butynyl)-3,5,5-trimethylcyclohex-2-en-1-one, 2-hydroxy-3,5,5-trimethyl-2-cyclohexen-1-one (2-hydroxyisophorone), 3-oxo-α-ionol, dehydrovomifoliol, and β-damascenone [18,71,79,81,84,100,101,105,106,107,108], were the compounds most frequently reported in heather honey.

### 3.10. Lavender Honey

Lavender honey is obtained when bees gather the nectar from the plants of the genus *Lavandula*, which is either cultivated or grown in the wild landscapes of the Mediterranean, mainly in France, Portugal and Spain (Table 11), and is characterized by a sweet, herbal infusion, floral, slightly medicinal licorice flavour [41]. The botanical source of this honey is mostly *Lavandula angustifolia*, *Lavandula latifolia*, *Lavandula stoechas* or from lavandin (*Lavandula angustifolia x latifolia*). Although other *Lavandula* species are not mentioned in the present studies, they may also contribute to the production of lavender honey, namely *Lavandula pedunculata* and *Lavandula luisieri* (“Rozeira” in Portuguese)—both very common in Portugal [109,110]. Some authors (Table 11) have pointed out linear aldehydes like hexanal and heptanal as characteristic of *Lavandula* honeys [49,52,79,98,111,112], although other studies showed marked differences in volatile profiles between the four monofloral types of this honey [41,79,99]. For instance, *Lavandula angustifolia* honey has a dominance of floral and honey-like smelling compounds like phenylacetaldehyde, phenylethanol, and β-damascenone [41], whereas *Lavandula latifolia* honey could be distinguished by the presence of 3,7-dimethyl-1,5,7-octatrien-3-ol and 3,5,5-trimethyl-2-cyclohexen-1-one, as well a high amount of 2,3-butanediol [99]. In *Lavandula stoechas* honey were mainly detected ethyl laurate and methyl stearate, phenylacetic acid, 3,5-dimethylphenol, benzyl alcohol, benzaldehyde and phenol [78], but no volatile compounds are characteristic of this honey [112]. Regarding lavandin honey, the mean contents, including lactones, namely γ-butyrolactone, pantolactone and γ-nonalactone, are higher than those in other lavender honeys [113]. Methyl alcohols were also abundant in lavender honey, especially 2-methyl-3-buten-2-ol [49,99,101], 3-methyl-2-buten-1-ol [98,99,112], and 2-methyl-2-buten-1-ol [49,97,99].

Based on the analysed data (Table 11), it was not possible to identify reliable markers that could differentiate the volatile profile of the four lavender honey types. For example, phenylacetaldehyde was common in both lavandin and *Lavandula angustifolia* honeys, whereas hexanoic and heptanoic acids were both identified in lavandin, *Lavandula stoechas and Lavandula latifolia* honeys [112,113].

### 3.11. Lime Tree Honey

The genus *Tilia* includes different species generally called lime trees, or linden for European species (Table 1). These species contribute to the production of lime tree honey, which has a taste which is sweet, bitter, medicinal, floral, woody and hay-like [41]. The described volatile compounds of linden tree honeys are included in all honeys obtained from different *Tilia* species, as these studies do not discriminate the species botanical source (Table 12). Monoterpene derivatives, namely rose oxides [71,75,103,114], *p*-methylacetophenone [52,71,75,85,103,111,114], carvacrol [41,69,71,75,85,103,111], *p*-cymene [41,70,71,85,103] and 8-*p*-menthen-1,2-diol [71,75] and α-terpinene [52,69,70,71,85,103,111], were the compounds most frequently reported in the literature. Aromatic hydrocarbons, such as dimethyl styrene [57,70,71], were also common constituents in *Tilia* honeys.

### 3.12. Orange Honey

Lilac aldehydes, lilac alcohols and linalool derivatives were the compounds most frequently reported in orange honeys (Table 13), characterized by a flavour which is very sweet and floral and has a slight bitterness [41,115]. Lilac aldehydes were identified in orange honeys from all ten countries with reported studies. Linalool derivatives such as *trans*-2,6-dimethyl-2,7-octadiene-1,6-diol, 2,6-dimethyl-3,7-octadiene-2,6-diol, *cis*-2,6-dimethyl-2,7-octadiene-1,6-diol, 1-hydroxylinalool were present in orange honeys from Greece, Spain and Palestine [87,92,116,117]. Dimethyl-1,5,7-octatrien-3-ol (hotrienol), also a linalool derivative, produced by the thermal dehydration of 2,6-dimethyl-3,7-octadiene-1,6-diol [118], was likewise present in several honey samples [41,79,83,100,115,119,120].

Methyl anthranilate, an ester frequently used in the fragrance and flavour industry due to its pleasant fruity odour [121] is frequently suggested as a volatile compound marker for *Citrus* honey [41,115,116]. Although the occurrence of methyl anthranilate is not among the five dominant compounds, it has been identified in ten out of twenty-three honeys.

### 3.13. Pine Honey and Honeydew

Pine honey, produced in Greece and Turkey, shows no incisive taste or aroma [122]. This type of honey is mainly obtained from honeydew secreted by the scale insect *Marchalina hellenica* that sucks the sap of pine trees, mainly of *Pinus brutia* Ten and *Pinus halepensis* Miller [122,123]. Nonanal, nonanol, decanal and octanal were the volatile compounds most frequently reported by three different studies on the profile of this honey (Table 14) [88,122,124].

### 3.14. Rape Honey

Rape honey is characterized by a sweet, musty and slightly fermented flavour [41]. The chemical composition of this type of honey, produced in nine different countries (Table 15) has been reported. Dimethyl disulfide was the most characteristic constituent detected in rape honeys produced in Denmark, France, Germany and Poland [52,71]. Besides this, there were several other compounds with variable presence depending on the honey origin: dimethyl trisulfide was observed for samples from Austria, Estonia and Germany, phenylacetic acid in samples from Estonia and Germany [41,105,125], both butyrolactone and pantolactone in Poland and Slovakia samples [70,71], benzyl alcohol in samples from Denmark, France, Germany and Poland [52,72], and benzoic acid in Estonia, Lithuania and Poland rape honey samples [55,72,105].

### 3.15. Raspberry Honey

Raspberry honey is not frequently commercially available and thus only two references were reported, concerning volatile compounds of this honey produced in Estonia and Slovakia (Table 16). This honey can be characterized by a large number of green notes and lack of honey notes [105]. According to Špánik et al. [70] 2-ethenylbuten-2-al, 3-methylhexane, 3-methylnonane, 3-pyridinemethanol, β-myrcene, cyclopentanemethanol, norbornane, and undecanal are characteristic of raspberry honey volatiles, although Seisonen et al. [105] didn´t find these compounds. It is clear that there is a need for further studies to ascertain the existence of a common volatile profile for this type of honey.

### 3.16. Rhododendron Honey

Rhododendron honey, characterized by having a woody and floral-fresh fruit aroma, originates from the species and natural hybrids spread in the Alps and Pyrenees, specifically *Rhododendron ferrugineum* L., *Rhododendron hirsutum* L. and their hybrid *Rhododendron* x *intermedium* [48]. In Turkey, this honey is obtained from the nectar of *Rhododendron ponticum* growing on the mountains of the eastern Black Sea and is usually known as “mad honey” or “toxic honey”, due to the presence of the toxic diterpenoids and grayanotoxins in the leaves, flowers, pollen and nectar of many *Rhododendron* species. However, these compounds were not detected in this honey type [126].

The description of volatiles in rhododendron honey, produced in five countries (Table 17) [76,77,79,87], did not highlight the presence of marker compounds or even common ones between them.

### 3.17. Rosemary Honey

*Rosmarinus officinalis* is cultivated in Mediterranean countries, particularly in Spain, contributing to honey production in different regions [78,100], with floral and fresh attributes [97]. Studies regarding the volatile fingerprinting of rosemary honey are mainly from Spain (Table 18), and despite the difficulty in identifying marker compounds for this type of honey, the alcohols 3-methyl-1-butanol, 3-methyl-3-buten-1-ol [79,97,99,100,101] and the norisoprenoid 4-oxoisophorone [52,79,97,100] were the most common constituents. Lilac aldehyde isomers were also identified in some rosemary honeys [79,97,100]. Benzaldehyde was also detected, but it can be considered a ubiquitous honey constituent.

### 3.18. Strawberry Tree Honey

Strawberry tree honey is produced in the south of Europe and has peculiar organoleptic characteristics, a distinct fragrance and a bitter aftertaste being particularly appreciated, although there are few data concerning its volatile composition [104,127] (Table 19).

Besides the usual volatiles identified in different honeys, such as alcohols, furan derivatives, esters or aldehydes, strawberry tree honey evidenced a high average content in norisoprenoids, which were common compounds between the honeys analysed from the three countries, highlighting 3,5,5-trimethyl-2-cyclohexen-1-one (α-isophorone), 3,5,5-trimethyl-3-cyclohexen-1-one (β-isophorone) and 3,5,5-trimethyl-cyclohex-2-ene-1,4-dione (4-oxoisophorone) (Table 19), which could be considered as marker compounds of these specific honeys.

### 3.19. Sunflower Honey

Sunflower is mainly cultivated for its oily seeds in several European countries, especially in eastern and southern ones, representing an important source of nectar and pollen to bees, contributing to the production of honey characterized by a floral-fresh fruit (fruity), warm and vegetal aroma [48].

The main volatile compounds from sunflower honeys, originating from seven countries (Table 20), were the alcohols 3-methyl-3-buten-1-ol, 3-methyl-2-butanol [52,69,111], and 1-butanol [69,76], and the monoterpene α-pinene [52,69,70,76,111].

### 3.20. Thyme Honey

Thyme honey is reported to possess a flavour with sweet, honey, lilac, bitter almond, thyme, violet, waxy, sour, ginger, caramel and rose characteristics [129]. Among the characteristic volatile compounds identified in more than one thyme honey sample (Table 21) are ethenyl phenylacetate and α-hydroxybenzenepropanoic acid [75,130] in honeys from Greece and Italy. Honey samples from Greece and Palestine evidenced 1,3-diphenyl-2-propanone, (3-methylbutyl)benzene, 3,4,5-trimethoxy benzaldehyde, 3,4-dimethoxy benzaldehyde, vanillin, and thymol [88,131], whereas 3-methyl-3-buten-1-ol, 2,3-butanedione, 2-methyl-3-buten-2-ol, 2,3-dihydro-4-methylfuran, 2-methyl-2-butenal, 2-butanol and linalool [49,97,99,101] were identified in thyme honeys from Spain. The volatile fingerprint of thyme honey exhibits several compounds that vary according to geographical origin, emphasizing the importance of the production area in the final volatile composition.

## 4. Conclusions

Nowadays, the marketing of monofloral honeys, particularly from a specific geographical region, assumes great importance on the part of the consumers, and the beekeeping sector is aware of this. Guaranteeing authenticity and differentiated quality in monofloral honeys reinforces the usefulness of identifying volatile compounds in order to provide the correct labelling of these honeys.

Although there are already studies on this topic, these are few and disperse, as the information is difficult to gather, as there are several variables to consider related to monofloral honey production such as geography, local flora, soil or climate and corresponding volatile analysis, including compound isolation and analytical procedures.

**Figure 1:**
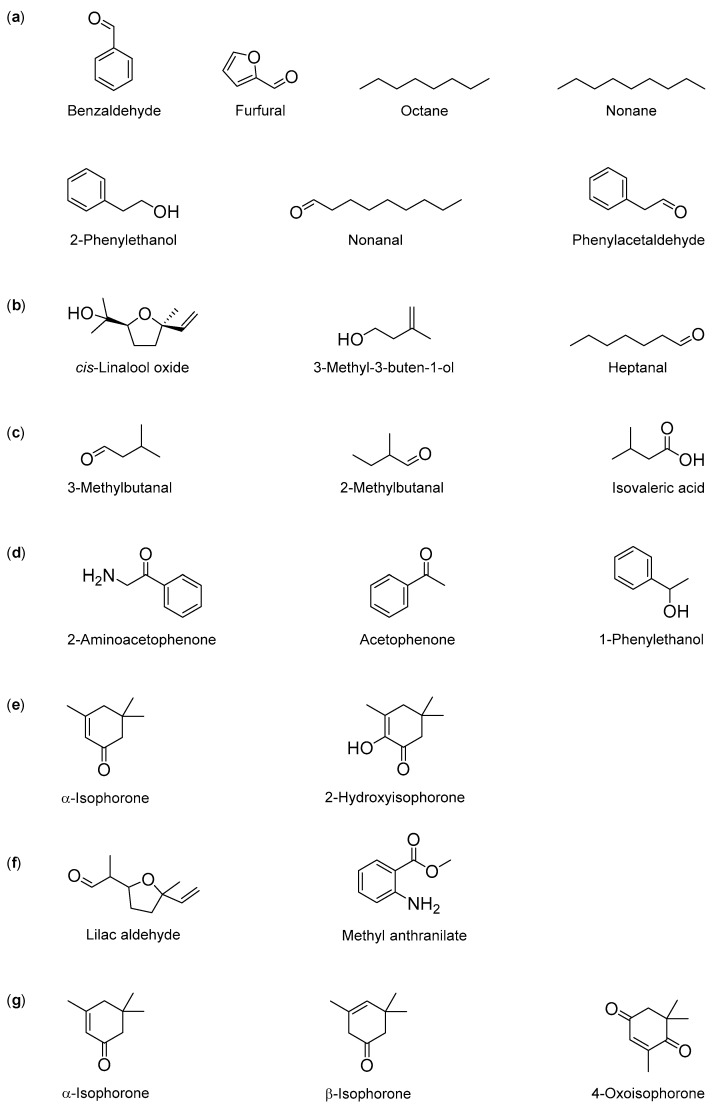
Chemical structures of volatile compounds identified in monofloral honeys: (**a**) Common volatile compounds to different types of monofloral honeys; Putative volatile marker compounds for (**b**) acacia honey, (**c**) buckwheat honey, (**d**) chestnut honey, (**e**) heather honey, (**f**) orange honey and (**g**) strawberry tree honey.

Aware of this variability, this review attempted to indicate as putative markers the volatile compounds that were most often reported in the several existing studies, from twenty selected monofloral honeys, highlighting the five dominant volatiles identified for each honey sample. However, these main components do not always correspond to markers for the analysed honey types, as other compounds, although present in smaller amounts, may be more often referred in a specific monofloral honey obtained from different countries. For this reason, some of the main volatile compounds could not be used as reliable markers, due to their ubiquity in different monofloral honeys (Figure 1), namely benzaldehyde, furfural, octane, nonane, 2-phenylethanol, nonanal or phenylacetaldehyde.

On the other hand, some specific volatile compounds may be used as markers for particular monofloral honeys, such as *cis*-linalool oxide, 3-methyl-3-buten-1-ol and heptanal for acacia honey, 3-methylbutanal, 2-methylbutanal and isovaleric acid (3-methylbutyric acid) for buckwheat honey, 2-aminoacetophenone, acetophenone and 1-phenylethanol for chestnut honey, α-isophorone and isophorone derivatives such as 2-hydroxyisophorone for heather honey, lilac aldehyde isomers and methyl anthranilate for orange honey and α-isophorone, β-isophorone and 4-oxoisophorone for strawberry tree honey (Figure 1). However, many more studies are needed to validate the importance of these compounds as volatile markers for the six mentioned monofloral honey types.

Despite the number of studies, the variability in the reported data did not allow for the recognition of marker compounds for some honey and honeydew types, such as those of clover, cotton, fir tree, pine, raspberry, rhododendron or thyme.

In contrast to the above, each of the monofloral honeys of dandelion, eucalyptus, lavender, lime tree, rape, rosemary and sunflower showed common compounds. Nevertheless, these compounds could not be considered reliable markers, since they did not occur in most of the samples of each honey type from different provenances.

Volatile markers for a specific monofloral honey from different regions may be rather different due to the presence of specific compounds in the flora of one country and their absence in another country. For a better understanding of this variability, comparative studies on the volatiles of local natural flora and the corresponding honey are desirable to better understand the relationship between both. Moreover, further research in this area should include both melissopalynological information and physicochemical data to understand to what extent volatile compounds can be used to classify with success the valuable monofloral honeys.

## Figures and Tables

**Table 1:** List of monofloral honey/honeydew types selected for volatile’s composition comparison. Common names are ordered alphabetically according to their main botanical sources.

Monofloral Honey/Honeydew Types	Scientific Name of the Dominant Botanical Source
Acacia	*Robinia pseudoacacia* L.
Buckwheat	*Fagopyrum esculentum* Moench
Chestnut	*Castanea sativa* Mill.
Clover	*Trifolium pratense* L., T *repens* L.
Cotton	*Gossypium hirsutum* L., *Gossympium* spp.
Dandelion	*Taraxacum officinale* Weber
Eucalyptus	*Eucalyptus* spp., *Eucalyptus melliodora* A. Cunn. ex Schauer, *E. leucoxylon* F.Muell, *E. camaldulensis* Dehnh.
Fir tree	*Abies* spp.
Heather	*Calluna vulgaris* (L.) Hull, *Erica multiflora* L., *Erica* spp., *Calluna* spp.
Lavender	*Lavandula angustifolia* Mill., *L. latifolia* Medik., *L. stoechas* L., *L. angustifolia* x *L. latifolia*, *Lavandula* spp.
Lime tree	*Tilia cordata* Mill., *T. europaea* L., *T. platyphyllos* Scop., *Tilia* spp.
Orange	*Citrus* spp.
Pine	*Pinus* spp.
Rape	*Brassica napus* L.
Raspberry	*Rubus idaeus* L.
Rhododendron	*Rhododendron* spp.
Rosemary	*Rosmarinus officinalis* L.
Strawberry tree	*Arbutus unedo* L.
Sunflower	*Helianthus annuus* L.
Thyme	*Thymus capitatus* L., *Thymus* spp.

**Table 2:** Acacia honey’s main volatiles, with reference to the country of origin, number of samples, isolation and analysis procedures and five main volatile components. Unless otherwise specified, the honeybee type was *A. mellifera*.

CHO	#	MPA	VIP	VA	Dominant Volatile Compounds	Ref.
Austria ^a^	1	nd	HS-SPME	GC-MS	Benzaldehyde, furfural, acetic acid, *cis*-linalool oxide (furanoid), 2-phenylethanol	[41]
China ^b^	1	nd	SPME	SPME/GC-MS	Ethanol, 2-octanone, furfural, 1-pentanol, ethyl acetate	[74]
Croatia ^a^	5	√	HD and USE	GC and GC-MS	HD: Phenylacetaldehyde, tetracosane, furfural, tricosane, *cis*-linalool oxide	[60]
					USE: Tetracosane, hexadecanoic acid, 1-hexadecanol, benzoic acid, 4-vinyl-2-methoxyphenol	
Czech Republic ^a^	10	√	Trap with Tenax TA	GC-MS	Ethanol, benzaldehyde, furfural, 2-methyl-3-buten-2-ol, 3-methyl-3-buten-1-ol	[69]
France ^b^	1	√	Trap with Tenax TA	GC-MS	Acetoin, acetone, furfural, benzaldehyde, 3,7-dimethyl-1,6-octadien-3-ol (linalool)	[52]
France ^a^	5	√	DHS	GC and GC-MS	Acetaldehyde, octane, 3-methyl-butanol, acetone, ethyl formate	[76]
Germany ^b^	2	√	Trap with Tenax TA	GC-MS	Acetoin, acetone, furfural, benzaldehyde, 3,7-dimethyl-1,6-octadien-3-ol (linalool)	[52]
Hungary ^a^	5	√	DHS	GC and GC-MS	Acetaldehyde, octane, 3-methyl-butanol, acetone, ethyl formate	[76]
Italy ^b^	4	√	Trap with Tenax TA	GC-MS	Acetoin, acetone, furfural, benzaldehyde, 6-methyl-5-hepten-2-one	[52]
Italy ^b^	3	√	HS-SPME	GC-MS	Acetic acid, ethanol, formic acid, isovaleric acid, furfural	[77]
Morocco ^a^	1	nd	HS-SPME	GC and GC-MS	*cis*-Linalool oxide, benzene acetaldehyde, furfural, *trans*-linalool oxide, benzaldehyde	[68]
Poland ^b^	8	nd	HS-SPME	GC and GC-MS	Benzaldehyde, nonanal, phenylacetaldehyde, furfural, heptanal	[71]
Poland ^b^	8	nd	SPME	GC-O-MS	Furfural, methylbutanal, 3-methylpentanal, 2-methylbutanol, 3-methylbutanol	[72]
Romania ^a^	10	√	Trap with Tenax TA	GC-MS	Ethyl acetate, ethanol, furfural, furanmethanol, hotrienol	[69]
Romania ^b^	30	√	SPE	GC-MS	Ethyl acetate, ethanol, furfural, benzaldehyde, *cis*-linalool oxide	[38]
Romania ^a^	1	√	DHS	GC and GC-MS	Acetaldehyde, octane, 3-methyl-butanol, acetone, ethyl formate	[76]
Romania ^a^	50	nd	SPME	GC-MS	Acetone (2-propanone), acetic acid, 2-furancarboxaldehyde (furfural), ethanol, linalool, benzaldehyde	[73]
Slovakia ^a^	17	nd	SPME	GC×GC-TOF-MS	Butan-2-one, alkanes (C6–C9) ^c^, nonanal, *cis*-linalool oxide, methyl ester of hexanoic and octanoic acid	[70]
Spain ^a^	5	√	L-N	GC and GC-MS	2-Phenylethanol, 2,3-pentanedione, 2-phenylacetaldehyde, methyl salicylate, ethyl phenylacetate	[78]
Spain ^a^	4	nd	SPME	GC-MS	Hotrienol, *cis*-linalool oxide, benzaldehyde, 3-methyl-butanoic acid, 3-methyl-3-buten-1-ol	[79]
Spain ^a^	10	√	Trap with Tenax TA	GC-MS	Hotrienol, acetone, ethanol, furfural, 2-butanol	[69]
Spain ^a^	1	√	DHS	GC and GC-MS	Acetaldehyde, octane, 3-methyl-butanol, acetone, ethyl formate	[76]

CHO: Country of honey origin. ^a^ *Robinia pseudoacacia*. ^b^ acacia. #: Number of samples studied. nd: no data. MPA: Melissopalynological analysis. √: MPA performed. VIP: Volatiles isolation procedure. VA: Volatiles analysis. Ref: References. HS-SPME: Headspace solid-phase microextraction. DHS: Dynamic headspace. L-N: Likens-Nickerson distillation extraction. GC: Gas chromatography. GC-MS: Gas chromatography-mass spectrometry. GC-O-MS: Gas chromatography-olfactometry-mass spectrometry. GC×GC-TOF-MS: Gas chromatography coupled to a time-of-flight mass spectrometer. HD: Hydrodistillation. SPE: Solid-phase extraction. USE: Ultrasound-assisted extraction. ^c^ C6–C9: Alkanes from hexadecane to nonadecane.

**Table 3:** Buckwheat honey’s main volatiles, with reference to the country of origin, number of samples, isolation and analysis procedures and five main volatile components. Unless otherwise specified, the honeybee type was *A. mellifera*.

CHO	#	MPA	VIP	VA	Dominant Volatile Compounds	Ref.
Italy ^a^	3	√	HS-SPME	GC-MS	Ethanol, isovaleric acid (3-methylbutanoic acid), acetic acid, formic acid, 3-methylbutanal	[77]
Italy ^a^	3	√	SPME	GC-MS	Isovaleric acid (3-methylbutanoic acid), linalool, furfural, 2-methylbutanal, 3-methylbutanal	[82]
Poland ^a^	1	nd	SPME	GC-MS	Isovaleric acid (3-methylbutanoic acid), 2-methyl-butanoic acid, benzaldehyde, dihydro-4-methyl-2(3H)-furanone, furfural	[81]
Poland ^a^	8	nd	SPME	GC-O-MS	Furfural, methylbutanal, pentanal, 2-methylbutanol, 3-methylbutanol	[72]
Poland ^a^	8	nd	HS-SPME	GC and GC-MS	Furfural, 2-methylbutanoic acid, isovaleric acid (3-methylbutanoic acid), 2-methylbutyraldehyde, 3-methylbutyraldehyde	[71]
United-Kingdom ^a^	1	nd	LLE	GC-MS	3-Methylbutanal, isovaleric acid (3-methylbutyric acid), 2,3-butanedione, 2-methylbutanal, butyric acid	[80]
United States of America ^a^	1	nd	LLE	GC-MS	Vanillin, 3-methylbutanal, isovaleric acid (3-methylbutyric acid), phenylacetaldehyde, 2,3-butanedione	[80]

CHO: Country of honey origin. ^a^ *Fagopyrum esculentum*. #: Number of samples studied. nd: no data. MPA: Melissopalynological analysis. √: MPA performed. VIP: Volatiles isolation procedure. VA: Volatiles analysis. Ref: References. HS-SPME: Headspace solid-phase microextraction. LLE: Liquid–liquid extraction. GC: Gas chromatography. GC-MS: Gas chromatography-mass spectrometry. GC-O-MS: Gas chromatography-olfactometry-mass spectrometry.

**Table 4:** Chestnut honey’s main volatiles, with reference to the country of origin, number of samples, isolation and analysis procedures and five main volatile components. Unless otherwise specified, the honeybee type was *A. mellifera*.

CHO	#	MPA	VIP	VA	Dominant Volatile Compounds	Ref.
Croatia ^a^	5	√	HD and USE	GC and GC-MS	HD: Tetracosane, phenylacetaldehyde, heneicosane, 4-aminoacetophenone, palmitic acid USE: Phenylacetic acid, 4-aminoacetophenone, 5-hydrohymethyl-2-furancarboxaldehyde, 2,3-dihydro-3,5-dihydroxy-6-methyl-4H-pyran-4-one (pyranone), cinnamyl alcohol	[60]
Croatia ^a^	1	nd	HS-SPME	GC-MS	Furfural, benzaldehyde, acetic acid, 2-methylbutanoic acid, benzyl alcohol	[41]
France ^a^	7	√	DHS	GC and GC-MS	Acetaldehyde, acetone, diacetyl, octane, hexane	[76]
France ^a^	nd	√	L-N	GC and GC-MS	Benzyl alcohol, furfuryl alcohol, 2-furaldehyde, 2-methylbutanoic acid, 2-phenylethanol	[85]
France ^a^	2	√	Trap with Tenax TA	GC-MS	3-Hydroxy-2-butanone (acetoin), furfural, 5-methylfurfural, 2-acetylfuran, 5-methyl-2(3H)-dihydrofuranone	[52]
Germany ^a^	2	√	Trap with Tenax TA	GC-MS	3-Hydroxy-2-butanone (acetoin), furfural, 5-methylfurfural, 2-acetylfuran, 5-methyl-2(3H)-dihydrofuranone	[52]
Greece ^a^	2	nd	HS-SPME	HS-SPME-GC-MS	Nonanal, furfural, decanal, nonanoic acid ethyl ester, α-isophorone	[88]
Italy ^a,b^	nd	√	L-N	GC-MS	3-Aminoacetophenone, benzyl alcohol, limonene or bornene, 2-phenylethanol, diethyl acetal	[86]
Italy ^a^	1	√	DHS	GC and GC-MS	Acetaldehyde, acetone, diacetyl (butanedione), octane, hexane	[76]
Italy ^a^	nd	√	L-N	GC and GC-MS	Benzyl alcohol, furfuryl alcohol, 2-furaldehyde, 2-methylbutanoic acid, 2-phenylethanol	[85]
Italy ^a^	3	nd	SPME	GC-MS	Nonanal, nonanol, benzaldehyde, camphor, acetophenone	[83]
Italy ^a^	5	√	Trap with Tenax TA	GC-MS	3-Hydroxy-2-butanone (acetoin), furfural, 5-methylfurfural, 2-acetylfuran, 5-methyl-2(3H)-dihydrofuranone	[52]
Italy ^a^	10	√	SPME	GC and GC-MS	*cis*-Linalool oxide, *trans*-linalool oxide, nonanal, nonanoic acid, aminoacetophenone	[75]
Portugal ^a^	2	√	HS-SPME	HS-SPME-GC-MS	Heptane, benzaldehyde, octane, nonanoic acid ethyl ester, nonanal	[84]
Spain ^a^	5	√	L-N	GC and GC-MS	2-Phenylethanol, phenol, 2-phenylacetamide, acetophenone, 2-aminoacetophenone	[78]
Spain ^a^	1	nd	SPME	GC-MS	1-Phenylethanol, 2-aminoacetophenone, acetophenone, 3-hexen-1-ol	[79]
Spain ^a^	10	nd	SPE	GC-MS	Phenylacetic acid, benzoic acid, 6-methyl-3,5-dihidroxy-2,3-dihydro-[4H] pyran-4-one (pyranone), 2-hydroxyacetophenone, cinnamic acid	[21]
Turkey ^a^	4	√	SPME	GC-MS	Phthalic acid, α-α-dimethylphenyl acetate, decane, phenylacetaldehyde, *p*-anisaldehyde	[87]

CHO: Country of honey origin. ^a^ *Castanea sativa*. ^b^ *A. mellifera carnica*. #: Number of samples studied. nd: no data. MPA: Melissopalynological analysis. √: MPA performed. VIP: Volatiles isolation procedure. VA: Volatiles analysis. Ref: References. HS-SPME: Headspace solid-phase microextraction. DHS: Dynamic headspace. L-N: Likens-Nickerson distillation extraction. GC: Gas chromatography. GC-MS: Gas chromatography-mass spectrometry. HD: Hydrodistillation. SPE: Solid-phase extraction. USE: Ultrasound-assisted extraction.

**Table 5:** Clover honey’s main volatiles, with reference to the country of origin, number of samples, isolation and analysis procedures and five main volatile components. Unless otherwise specified, the honeybee type was *A. mellifera*.

CHO	#	MPA	VIP	VA	Dominant Volatile Compounds	Ref.
Australia ^a^	2	nd	LLE	GC and GC-MS	Furfuraldehyde, 5-hydroxymethylfurfural, 3-hydroxypentan-2-one, methylmethoxyfuran, ionol	[89]
Canada ^a^	2	√	DHS	GC and GC-MS	Acetaldehyde, ethyl formate, octane, acetone, ethanol	[76]
Croatia ^b,c^	5	√	HS-SPME and USE	GC and GC-MS	HS-SPME: Lilac aldehyde (isomer II), phenylacetaldehyde, benzaldehyde, lilac aldehyde (isomer III), lilac aldehyde (isomer I) USE: *cis*-Octadec-9-en-1-ol, nonadecane, hexadecan-1-ol, octadecan-1-ol, hexadecenoic acid	[91]
France ^a^	1	√	DHS	GC and GC-MS	Acetaldehyde, ethyl formate, octane, acetone, ethanol	[76]
Lithuania ^a^	1	√	SPME	GC-MS	Nonanal, 2-methylbutanenitrile, 2-methylpropanenitrile, 2-methyl propanoic acid, benzaldehyde	[55]
New Zealand ^a^	8	nd	LLE	GC and GC-MS	Dimethyl 2-decenedioate, dimethyl succinate, methyl 2-hydroxy-3-phenylpropionate, methyl palmitate, dimethyl decanedioate	[90]
New Zealand ^a^	1	√	DHS	GC and GC-MS	Acetaldehyde, ethyl formate, octane, acetone, ethanol	[76]

CHO: Country of honey origin. ^a^ *Trifolium repens*. ^b^ *Trifolium pratense*. ^c^ *A. mellifera carnica*. #: Number of samples studied. nd: no data. MPA: Melissopalynological analysis. √: MPA performed. VIP: Volatiles isolation procedure. VA: Volatiles analysis. Ref: References. HS-SPME: Headspace solid-phase microextraction. DHS: Dynamic headspace. GC: Gas chromatography. GC-MS: Gas chromatography-mass spectrometry. LLE: Liquid–liquid extraction. USE: Ultrasound-assisted extraction.

**Table 6:** Cotton honey’s main volatiles, with reference to the country of origin, number of samples, isolation and analysis procedures and five main volatile components. Unless otherwise specified, the honeybee type was *A. mellifera*.

CHO	#	MPA	VIP	VA	Dominant Volatile Compounds	Ref.
Greece ^a^	7	nd	HS-SPME	GC-MS	Phenylacetic acid, phenylethyl alcohol, oleic acid, palmitic acid, tricosane	[93]
Greece ^b^	3	nd	HS-SPME	HS-SPME-GC-MS	Octane, nonanal, decanal, nonane, nonanoic acid ethyl ester	[88]
Palestine ^a^	3	nd	HS-SPME	HS-SPME-GC-MS	Phenylacetaldehyde, phenylethyl alcohol, 2-ethyl hexanoic acid, nonanoic acid, 5-hydroxymethyl 2-furancarboxaldehyde	[92]
Turkey ^a^	2	√	SPME	GC-MS	Nonanal, phenylacetaldehyde, phenylethyl alcohol, safranal	[87]

CHO: Country of honey origin. ^a^ *Gossypium hirsutum*. ^b^ *Gossipium* spp. #: Number of samples studied. nd: no data. MPA: Melissopalynological analysis. √: MPA performed. VIP: Volatiles isolation procedure. VA: Volatiles analysis. Ref: References. HS-SPME: Headspace solid-phase microextraction. GC: Gas chromatography. GC-MS: Gas chromatography-mass spectrometry.

**Table 7:** Dandelion honey’s main volatiles, with reference to the country of origin, number of samples, isolation and analysis procedures and five main volatile components. Unless otherwise specified, the honeybee type was *A. mellifera*.

CHO	#	MPA	VIP	VA	Dominant Volatile Compounds	Ref.
Austria ^a^	1	nd	HS-SPME	GC-MS	2-Methylbutanoic acid, hotrienol, 2-methylpropanoic acid, benzylnitrile, 2-methylpentanoic acid	[41]
France ^a^	1	√	DHS	GC and GC-MS	Octane, ethyl formate, ethanol, acetone, acetaldehyde	[76]
Italy ^a^	4	√	SPME	GC and GC-MS	Hexanenitrile, ethanol, pentanenitrile, phenylacetonitrile, furfuryl *n*-butyrate	[75]
Spain ^a^	1	nd	SPME	GC-MS	2-Methylpropanenitrile, 2-methyl-butanenitrile, 3-methyl-butanenitrile, 2-butenenitrile (*cis*- or *trans*-isomers), 3-butenenitrile	[79]
Sweden ^a^	1	√	HS-SPME, USE, SPE	GC-MS	HS-SPME: 3-Methylpentanenitrile, phenylacetonitrile, benzaldehyde, 3-hydroxy-4-phenylbutan-2-one, *trans*-β-damascenone SPE: Phenylacetic acid, dehydrovomifoliol, docosane, 3-hydroxy-4-phenylbutan-2-one, *cis*-octadec-9-en-1-ol USE: Phenylacetic acid, dehydrovomifoliol, 3-methylpentanoic acid, 3-hydroxy-4-phenylbutan-2-one, *cis*-octadec-9-en-1-ol	[94]

CHO: Country of honey origin. ^a^ *Taraxacum officinale*. #: Number of samples studied. nd: no data. MPA: Melissopalynological analysis. √: MPA performed. VIP: Volatiles isolation procedure. VA: Volatiles analysis. Ref: References. HS-SPME: Headspace solid-phase microextraction. DHS: Dynamic headspace. GC: Gas chromatography. GC-MS: Gas chromatography-mass spectrometry. SPE: Solid-phase extraction. USE: Ultrasound-assisted extraction.

**Table 8:** Eucalyptus honey’s main volatiles, with reference to the country of origin, number of samples, isolation and analysis procedures and five main volatile components. Unless otherwise specified, the honeybee type was *A. mellifera*.

CHO	#	MPA	VIP	VA	Dominant Volatile Compounds	Ref.
Australia ^a^	1	nd	LLE	GC and GC-MS	3-Hydroxy-2-butanone (acetoin), 5-hydroxymethyl-2-furaldehyde (HMF), hexenyl butyrate (II), 3-hydroxypentan-2-one, hexenyl butyrate (I)	[89]
Australia ^b^	2	√	DHS	GC and GC-MS	Octane, diacetyl, acetaldehyde, dimethylsulphide, acetone	[76]
Australia ^b^	7	√	L-N	GC and GC-MS	3-Hydroxy-2-butanone (acetoin), 3-hexanol, 3,4-hexanedione, phenol, octane	[98]
Australia ^c^	7	nd	LLE	GC and GC-MS	*Levo*-butane-2,3-diol, 3-hydroxybutan-2-one (acetoin), 18-hydroxyoleic acid lactone, 3-oxo-α-ionone, dehydrovomifoliol	[95]
Australia ^a^	6	nd	LLE	GC and GC-MS	18-Hydroxyoleic acid lactone, *levo*-butane-2,3-diol, dehydrovomifoliol, *meso*-butane-2,3-diol, 5-(hydroxymethyl)-2-furfural (HMF)	[95]
Italy ^b^	1	√	L-N	GC and GC-MS	3-Hydroxy-2-butanone (acetoin), 3-hexanol, 3,4-hexanedione, phenol, octane	[98]
Italy ^b^	2	√	Trap with Tenax TA	GC-MS	3-Hydroxy-2-butanone (acetoin), 2-pentanone, 1-octene, 2,3-pentanedione	[52]
Italy ^d^	3	nd	SPME	GC-MS	Nonanol, nonanal, nonanoic acid, 5-hexen-2-ol, 2,3-dimethyl-5-hexen-2-ol	[83]
Italy ^b^	8	√	SPME	GC and GC-MS	4-(3-Hydroxy-1-butenyl)-3,5,5-trimethyl-2-cyclohexen-1-one (3-oxo-α-ionol), nonanoic acid, 2-phenylethanol, nonanal, benzylalcohol	[75]
Italy ^d^	1	nd	DHS, SBSE, SPME	GC-MS	DHS: Nonanal, nonan-1-ol, benzyl alcohol, benzaldehyde, octane SBSE: Nonan-1-ol, decanal, nonanoic acid, nonanal, octane SPME: Nonanal, nonan-1-ol, octane, nonanoic acid, *p*-cymene	[62]
Morocco ^b^	1	nd	HS-SPME	GC and GC-MS	*cis*-Linalool oxide, *trans*-linalool oxide, hotrienol, furfural, dimethyl sulphide	[68]
Palestine ^d^	3	nd	HS-SPME	HS-SPME-GC-MS	Phenylacetaldehyde, tetradecane, 2-ethyl hexanoic acid, pentadecane, nonanoic acid	[92]
Portugal ^b^	4	√	HS-SPME	HS-SPME-GC-MS	Heptane, octane, linalool L, octanoic acid ethyl ester, nonanoic acid ethyl ester	[84]
Spain ^b^	1	√	DHS	GC and GC-MS	Octane, diacetyl, acetaldehyde, dimethylsulphide, acetone	[76]
Spain ^b^	2	√	L-N	GC and GC-MS	3-Hydroxy-2-butanone (acetoin), 3-hexanol, 3,4-hexanedione, phenol, octane	[98]
Spain ^b^	1	√	Trap with Tenax TA	GC-MS	3-Hydroxy-2-butanone (acetoin), 2-pentanone, 1-octene, 2,3-pentanedione	[52]
Spain ^b^	4	nd	SPME	GC-MS	2,3-Butanediol, 3-hydroxy-2-butanone (acetoin), acetic acid, ethanol, 1-hydroxy-2-propanone	[99]
Spain ^b^	10	√	L-N	GC and GC-MS	3-Hydroxy-2-butanone (acetoin), 1-hexyl alcohol, furfuryl propionate, 2,3-pentanedione, 2-acetyl-5-methylfuran	[78]
Spain ^b^	21	nd	HS-SPME	GC-MS	2,3-Butanedione, 3-hydroxy-2-butanone (acetoin), *cis*-linalool oxide (furanoid), 3-methyl-1-butanol, benzaldehyde	[100]
Spain ^e^	5	nd	SPE	GC-MS	Hydroxymethylfurfural, 6-methyl-2-methoxypyrazine, methyl furoate, 3-oxo-α-ionone, 2-methyl butanoic acid	[96]
Spain ^b^	3	nd	P&T	P&T-GC-MS	2,3-Butanedione, octane, dimethylsulfide, heptane, acetonitrile	[101]
Spain ^b^	4	nd	SPME	GC-MS	Nonanoic acid, octanoic acid, 1-nonanol, phenylacetaldehyde, 3-methyl-butanoic acid	[79]
Spain ^b^	10	nd	SDE	GC-MS	Phenylacetaldehyde, 3-hydroxy-2-butanone (acetoin), furfural, 3-hydroxy-5-methyl-2-hexanone, nonanoic acid	[97]
Turkey ^b^	2	√	SPME	GC-MS	Nonanal, ethylphenyl acetate, phenethyl alcohol	[87]

CHO: Country of honey origin. ^a^ *Eucalyptus melliodora* (Yellow box). ^b^ *Eucalyptus* spp. ^c^ *Eucalyptus leucoxylon* (Blue gum). ^d^ *Eucalyptus camaldulensis*. ^e^ *Eucalyptus globulus*. #: Number of samples studied. nd: no data. MPA: Melissopalynological analysis. √: MPA performed. VIP: Volatiles isolation procedure. VA: Volatiles analysis. Ref: References. HS-SPME: Headspace solid-phase microextraction. DHS: Dynamic headspace. L-N: Likens-Nickerson distillation extraction. GC: Gas chromatography. GC-MS: Gas chromatography-mass spectrometry. LLE: Liquid–liquid extraction. SDE: Simultaneous distillation-extraction. P&T: Purge and trap concentrator. SBSE: Stir bar sorptive extraction.

**Table 9:** Main volatiles of fir tree honey and honeydew, with reference to the country of origin, number of samples, isolation and analysis procedures and five main volatile components. Unless otherwise specified, the honeybee type was *A. mellifera*.

CHO	#	MPA	VIP	VA	Dominant Volatile Compounds	Ref.
Austria ^a^	1	nd	HS-SPME	GC-MS	Lilac aldehyde D, acetic acid, benzaldehyde, 2-methylbutanoic acid, furfural	[41]
Croatia ^b^	3	√	HS-SPME	GC-MS	Acetonitrile, methyl-2-buten-1-ol, *n*-hexanol, 1-propyne, 2-furanmethanol (furfuryl alcohol)	[103]
France ^a^	7	√	DHS	GC and GC-MS	Acetaldehyde, acetone, ethyl formate, 3-methyl-butanol, octane	[76]
Greece ^c^	2	nd	HS-SPME	HS-SPME-GC-MS	Nonane, 1-nonanol, decanoic acid ethyl ester, octanal, *cis*-5-methyl-4-nonene	[102]
Greece ^a^	3	nd	HS-SPME	HS-SPME-GC-MS	Nonanoic acid ethyl ester, octanoic acid ethyl ester, decanoic acid ethyl ester, nonanal, nonane	[88]
Spain ^a^	nd	nd	SPME	GC-MS	Butanoic acid, methyl butyrate, α-pinene, α-phellandrene, α-terpinene	[104]

CHO: Country of honey origin. ^a^ *Abies* spp. honey. ^b^ *Abies alba* honeydew. ^c^ *Abies* spp. honeydew. #: Number of samples studied. nd: no data. MPA: Melissopalynological analysis. √: MPA performed. VIP: Volatiles isolation procedure. VA: Volatiles analysis. Ref: References. HS-SPME: Headspace solid-phase microextraction. DHS: Dynamic headspace. GC: Gas chromatography. GC-MS: Gas chromatography-mass spectrometry.

**Table 10:** Heather honey’s main volatiles, with reference to the country of origin, number of samples, isolation and analysis procedures and five main volatile components. Unless otherwise specified, the honeybee type was *A. mellifera*.

CHO	#	MPA	VIP	VA	Dominant Volatile Compounds	Ref.
Belgium ^a^	nd	√	L-N	GC and GC-MS	Acetoin, 4-(3-oxobut-1-enylidene)-3,5,5-trimethyl-cyclohex-2-en-1-one, α-isophorone, benzylalcohol, 2-phenylethanol	[108]
England ^b^	3	√	Trap with Tenax TA	GC-MS	Acetoin, 1-penten-3-ol, byciclo 2,2,2-octan-1-ol-4-methyl, 2-methylpropanoic acid, phenylacetaldehyde	[52]
Estonia ^a^	2	√	SPME	GC-MS and GC-O	Phenylacetaldehyde, hydrocinnamic acid, butyric acid, dimethyl trisulphide, hexyl hexanoate	[105]
France ^a^	nd	√	L-N	GC and GC-MS	Acetoin, 4-(3-oxobut-1-enylidene)-3,5,5-trimethyl-cyclohex-2-en-1-one, α-isophorone, benzylalcohol, 2-phenylethanol	[108]
France ^c^	nd	√	L-N	GC and GC-MS	*p*-Anisaldehyde (4-methoxybenzaldehyde), 1-methoxy-4-propyl-benzene, *p*-anisic acid (4-methoxybenzoic acid), 2-furaldehyde, cinnamyl alcohol	[108]
France ^b^	1	√	Trap with Tenax TA	GC-MS	Acetoin, byciclo 2,2,2-octan-1-ol-4-methyl, phenylacetaldehyde, ethanol, 2-methyl-1-propanol	[52]
Germany ^a^	nd	√	L-N	GC and GC-MS	Acetoin, 4-(3-oxobut-1-enylidene)-3,5,5-trimethyl-cyclohex-2-en-1-one, α-isophorone, benzylalcohol, 2-phenylethanol	[108]
Germany ^b^	2	√	Trap with Tenax TA	GC-MS	Acetoin, byciclo 2,2,2-octan-1-ol-4-methyl, 2-methylpropanoic acid, phenylacetaldehyde, ethanol	[52]
Greece ^c^	nd	√	L-N	GC and GC-MS	*p*-Anisaldehyde (4-methoxybenzaldehyde), 1-methoxy-4-propyl-benzene, *p*-anisic acid (4-methoxybenzoic acid), 2-furaldehyde, cinnamyl alcohol	[108]
Italy ^c^	nd	√	L-N	GC and GC-MS	*p*-Anisaldehyde (4-methoxybenzaldehyde), 1-methoxy-4-propyl-benzene, *p*-anisic acid (4-methoxybenzoic acid), 2-furaldehyde, cinnamyl alcohol	[108]
Netherlands ^b^	2	√	Trap with Tenax TA	GC-MS	Acetoin, byciclo 2,2,2-octan-1-ol-4-methyl, 2-methylpropanoic acid, phenylacetaldehyde, ethanol	[52]
New Zealand ^a^	3	√	LLE	GC and GC-MS	4-hydroxy-4-(3-oxo-l-butenyl)-3,5,5-trimethylcyclohex-2-en-1-one, 4-(3-oxo-l-butynyl)-3,5,5-trimethylcyclohex-2-en-1-one, methyl myristate, 3,5,5-trimethylcyclohex-2-ene-1,4-dione (4-oxoisophorone), α-isophorone	[107]
Norway ^a^	nd	√	L-N	GC and GC-MS	Acetoin, 4-(3-oxobut-1-enylidene)-3,5,5-trimethyl-cyclohex-2-en-1-one, α-isophorone, benzylalcohol, 2-phenylethanol	[108]
Poland ^b^	1	nd	SPME	GC-MS	Benzene acetaldehyde, 1,2,4-trimethyl-5-benzene (cumene, 2,4,5-trimethyl-), benzaldehyde, 3,4,5-trimethylphenol, isobutyl phthalate	[81]
Poland ^b^	8	nd	HS-SPME	GC and GC-MS	3,4,5-Trimethylphenol, phenylacetic acid, benzoic acid, β-damascenone, 3-oxodamascenone	[71]
Portugal ^d^	1	√	HS-SPME	HS-SPME-GC-MS	Heptane, hotrienol, benzaldehyde, 2-furancarboxaldehyde, *cis*-linalool oxide	[84]
Spain ^d,e^	33	nd	HS-SPME	GC-MS	3-Methyl-1-butanol, benzaldehyde, benzene acetaldehyde, 3-methyl-3-buten-1-ol, *cis*-linalool oxide (furanoid)	[100]
Spain ^b^	1	nd	P&T	P&T-GC-MS	Acetonitrile, dimethylsulfide, heptane, 2-methyl-3-buten-2-ol, 2-methyl-1-propanol	[101]
Spain ^b^	5	nd	SDE	GC-MS	Phenylacetaldehyde, propylanisole, furfural, 2-phenylethanol, benzyl alcohol	[97]
Spain ^b^	2	nd	SPME	GC-MS	2-Phenylethanol, benzyl alcohol, hotrienol, benzaldehyde, 4-oxoisophorone	[79]
Spain ^f^	6	√	SPME	GC-MS	*trans*-Linalool oxide, *p*-menthane-1,8-diol, safranal, limonene, α-pinene	[18]
United Kingdom ^a^	nd	√	L-N	GC and GC-MS	Acetoin, 4-(3-oxobut-1-enylidene)-3,5,5-trimethyl-cyclohex-2-en-1-one, α-isophorone, benzylalcohol, 2-phenylethanol	[108]

CHO: Country of honey origin. ^a^ *Calluna vulgaris*. ^b^ Heather. ^c^ *Erica arborea*. ^d^ *Erica* spp. ^e^ *Calluna* spp. ^f^ *Erica multiflora*. #: Number of samples studied. nd: no data. MPA: Melissopalynological analysis. √: MPA performed. VIP: Volatiles isolation procedure. VA: Volatiles analysis. Ref: References. HS-SPME: Headspace solid-phase microextraction. LLE: Liquid–liquid extraction. P&T: Purge and trap concentrator. L-N: Likens-Nickerson distillation extraction. GC: Gas chromatography. GC-O: Gas chromatography-olfactometry. GC-MS: Gas chromatography-mass spectrometry.

**Table 11:** Lavender honey’s main volatiles, with reference to the country of origin, number of samples, isolation and analysis procedures and five main volatile components. Unless otherwise specified, the honeybee type was *A. mellifera*.

CHO	#	MPA	VIP	VA	Dominant Volatile Compounds	Ref.
Croatia ^a^	1	nd	HS-SPME	GC-MS	*cis*-Thujone, camphor, 2-phenethyl acetate, 1,8-cineole, furfural	[41]
France ^b^	12	√	DHS	GC and GC-MS	Octane, acetaldehyde, caproaldehyde, ethyl formate, diacetyl	[76]
France ^b^	9	√	L-N	GC and GC-MS	*n*-Hexanol, phenylacetaldehyde, coumarin, 2-phenylethanol, phenol	[98]
France ^b^	1	√	Trap with Tenax TA	GC-MS	Acetoin, hexanal, heptanal, 1-hexanol, furfural	[52]
France ^c^	6	√	L-N	GC and GC-MS	*n*-Hexanol, phenylacetaldehyde, *n*-nonanal, *n*-hexanal, 2-phenylethanol	[112]
France ^a^	4	√	L-N	GC and GC-MS	*n*-Hexanol, *n*-nonanal, phenylacetaldehyde, *n*-hexanal, 2-phenylethanol	[112]
Portugal ^b^	1	√	Trap with Tenax TA	GC-MS	Acetoin, ethanol, 2-methyl-1-propanol, 3-methyl-1-butanol, 3-methyl-3-buten-1-ol	[52]
Portugal ^d^	5	√	L-N	GC and GC-MS	Hexanoic acid, 2-phenylethanol, phenylacetaldehyde, n-nonanal, pyridine	[112]
Spain ^b^	1	√	DHS	GC and GC-MS	Octane, acetaldehyde, caproaldehyde, ethyl formate, diacetyl	[76]
Spain ^b^	1	√	L-N	GC and GC-MS	*n*-Hexanol, phenylacetaldehyde, coumarin, 2-phenylethanol, phenol	[98]
Spain ^e^	4	nd	SPME	GC-MS	2,3-Butanediol, dimethyl sulfide, acetic acid, 1-hydroxy-2-propanone, 3-methyl-3-buten-1-ol	[99]
Spain ^d^	5	√	L-N	GC and GC-MS	Ethyl laurate, phenol, 3-phenylpropionate, 2-phenylethanol, dimethyldisulphide	[78]
Spain ^f^	1	nd	P&T	P&T-GC-MS	Heptane, dimethylsulfide, 2-methyl-3-buten-2-ol, 2-methyl-1-propanol, octane	[101]
Spain ^f^	2	nd	SPME	GC-MS	Benzaldehyde, *n*-hexanol, phenylacetaldehyde, hexanal, heptanal	[79]
Spain ^b^	7	nd	SDE	GC-MS	Phenylacetaldehyde, furfural, hotrienol, 2-phenylethanol, hexanol	[97]
Spain ^c^	10	√	SPE	GC-MS	Triethylenglycol, 2,6-dimethyl-3,7-octadien-2,6-diol, benzoic acid, hexadecanoic acid, benzenacetic acid	[113]
Spain ^e^	10	√	SPE	GC-MS	Triethylenglycol, hydroxymethylfurfural, 2,6-dimethyl-3,7-octadien-2,6-diol, hexadecanoic acid, coumarin	[113]
Spain ^b^	36	√	Trap with Tenax TA	GC-MS	Ethanol, heptanal, 2-butanol, 3-methylbutanal, 2-methyl-1-butanol	[49]

CHO: Country of honey origin. ^a^ *Lavandula angustifolia*. ^b^ Lavender. ^c^ Lavandin (*Lavandula angustifolia x Lavandula latifolia*). ^d^ *Lavandula stoechas*. ^e^ *Lavandula latifolia*. ^f^ *Lavandula* spp. #: Number of samples studied. nd: no data. MPA: Melissopalynological analysis. √: MPA performed. VIP: Volatiles isolation procedure. VA: Volatiles analysis. Ref: References. HS-SPME: Headspace solid-phase microextraction. DHS: Dynamic headspace. P&T: Purge and trap concentrator. SDE: Simultaneous distillation-extraction. SPE: Solid-phase extraction. L-N: Likens-Nickerson distillation extraction. GC: Gas chromatography. GC-MS: Gas chromatography-mass spectrometry.

**Table 12:** Lime tree honey’s main volatiles, with reference to the country of origin, number of samples, isolation and analysis procedures and five main volatile components. Unless otherwise specified, the honeybee type was *A. mellifera*.

CHO	#	MPA	VIP	VA	Dominant Volatile Compounds	Ref.
China ^a^	1	√	SDE	GC and NMR	*trans*-β-Damascenone, 4-vinylguaiacol, linalool, *cis*-rose oxide, 2-acetyl-1 -pyrroline	[114]
China ^b^	1	nd	SPME	SPME/ GC-MS	Furfural, ethanol, 2-octanone, 2-phenylacetaldehyde, 2,3-butanediol	[74]
Croatia ^c^	5	√	HS-SPME	GC-MS	*trans*-2-Caren-4-ol, terpinene, rose oxide, 4-methyl-1-(1-methylethyl)-3-cylohexen-1-ol (4-terpinenol), 1-(4-methylphenyl)-ethanone (*p*-methylacetophenone)	[103]
Croatia ^d^	1	nd	HS-SPME	GC-MS	1-Methyl-4-(1-methylethenyl)-benzene, 2,3-dehydro-1,8-cineole, acetic acid, α-terpinen-4-ol, benzaldehyde	[41]
Czech Republic ^c^	10	√	Trap with Tenax TA	GC-MS	Ethyl acetate, furfural, carvacrol, acetone, hotrienol	[69]
France ^c^	10	√	L-N	GC and GC-MS	2-Phenylethanol, benzyl alcohol, 2-furaldehyde, 8-*p*-menthene-1,2-diol, trimethoxybenzene isomer	[85]
Germany ^e^	2	√	Trap with Tenax TA	GC-MS	2-Pentanone, acetoin, furfural, methyl isopropylbenzene, dimethylstyrene	[52]
Italy ^e^	11	√	SPME	GC and GC-MS	8-*p*-Menthen-1,2-diol, dimethylstyrene, carvacrol, 2-phenylethanol, 2-(*p*-methoxyphenyl)ethanol	[75]
Netherlands ^e^	2	√	Trap with Tenax TA	GC-MS	2-Pentanone, acetoin, furfural, methyl isopropylbenzene, dimethylstyrene	[52]
Poland ^b^	8	nd	HS-SPME	GC and GC-MS	Dimethylstyrene, furfural, methylstyrene, *p*-methylacetophenone, 8-*p*-menthen-1,2-diol	[71]
Romania ^a^	1	√	SDE	GC and NMR	*trans*-β-Damascenone, phenylacetaldehyde, *p*-anisaldehyde, methional, 2-acetyl-1-pyrroline	[114]
Romania ^c^	10	√	Trap with TenaxTA	GC-MS	Ethanol, ethyl acetate, furfural, acetone, 2-methyl-3-buten-2-ol	[69]
Romania ^f^	12	√	SPE	GC-MS	Ethyl acetate, furfural, carvacrol, acetone, ethanol	[111]
Slovakia ^a^	6	nd	SPME	GC×GC-TOF-MS	*trans*-3(10)-Caren-2-ol, 4-terpineol, 2,3-butanedione, 4-oxoisophorone, *p*-cymene	[70]

CHO: Country of honey origin. ^a^ *Tilia cordata*. ^b^ Linden tree. ^c^ *Tilia* spp. ^d^ *Tilia platyphyllos*. ^e^ Lime tree. ^f^ *Tilia* x *europaea* (*Tilia cordata* x *Tilia platyphyllos*) #: Number of samples studied. nd: no data. MPA: Melissopalynological analysis. √: MPA performed. VIP: Volatiles isolation procedure. VA: Volatiles analysis. Ref: References. HS-SPME: Headspace solid-phase microextraction. LLE: Liquid-liquid extraction. L-N: Likens-Nickerson distillation extraction. GC×GC-TOF-MS: Gas chromatography coupled to a time-of-flight mass spectrometer. NMR: Nuclear magnetic resonance. GC: Gas chromatography. GC-MS: Gas chromatography-mass spectrometry.

**Table 13:** Orange honey’s main volatiles, with reference to the country of origin, number of samples, isolation and analysis procedures and five main volatile components. Unless otherwise specified, the honeybee type was *A. mellifera*.

CHO	#	MPA	VIP	VA	Dominant Volatile Compounds	Ref.
Croatia ^a^	1	nd	HS-SPME	GC-MS	Furfural, lilac aldehyde A, lilac aldehyde B, 2,3-butanediol, acetic acid	[41]
Egypt ^b^	7	√	HS-SPME	HS-SPME-GC-MS	Lilac aldehyde (isomer III), lilac aldehyde (isomer I), furfural, dill ether, ethyl decanoate	[121]
France ^b^	4	√	DHS	GC and GC-MS	Acetaldehyde, diacetyl, acetone, ethyl formate, dimethylsulphide	[76]
Greece ^b^	2	nd	USE	GC-MS	*trans*-2,6-Dimethyl-2,7-octadiene-1,6-diol, 2,6-dimethyl-3,7-octadiene-2,6-diol, 3,7-dimethyl-1,5,7-octatrien-3-ol (hotrienol), cis-2,6-dimethyl-2,7-octadiene-1,6-diol, *m*- (or *p*-) xylene	[119]
Greece ^b^	nd	nd	HS-SPME	GC-MS	Lilac aldehyde (isomer II), lilac aldehyde (isomer I), lilac aldehyde (isomer III), limonene, methyl anthranilate	[115]
Greece ^b^	16	√	HS-SPME	HS-SPME-GC-MS	Lilac aldehyde (isomer III), lilac aldehyde (isomer II), lilac aldehyde (isomer I), nonanal, benzene acetaldehyde	[121]
Greece ^b^	5	nd	HS-SPME	HS-SPME-GC-MS	5-Isoprenyl-2-methyl-2-vinyl-tetrahydrofuran, α-4-dimethyl-3-cyclohexene-1-acetaldehyde, dill ether, octane, lilac aldehyde (IV)	[88]
Italy ^a^	3	nd	SPME	GC-MS	Dimethyl-1,5,7-octatrien-3-ol (hotrienol), methyl anthranilate, dimethylsulfide	[83]
Italy ^b^	5	√	SPME	GC and GC-MS	Limonene diol, methyl anthranilate, 2-phenylethanol, lilac alcohol, lilac aldehyde	[75]
Italy ^b^	nd	nd	HS-SPME	GC-MS	Lilac aldehyde (isomer II), lilac aldehyde (isomer I), lilac aldehyde (isomer III), limonene, methyl anthranilate	[115]
Mexico ^b^	3	√	DHS	GC and GC-MS	Acetaldehyde, diacetyl, acetone, ethyl formate, dimethylsulphide	[76]
Morocco ^b^	6	√	HS-SPME	HS-SPME-GC-MS	Lilac aldehyde (isomer III), furfural, acetic acid, dill ether, herboxide isomer II	[121]
Palestine ^b^	3	nd	HS-SPME	HS-SPME-GC-MS	Phenylacetaldehyde, phenylethylalcohol, 2-ethyl hexanoic acid, nonanoic acid, benzoic acid	[92]
Spain ^b^	2	√	DHS	GC and GC-MS	Acetaldehyde, diacetyl, acetone, ethyl formate, dimethylsulphide	[76]
Spain ^b^	4	nd	SPME	GC-MS	Furfural, acetic acid, acetone, 1-hydroxy-2-propanone, 2-amino methyl benzoate	[99]
Spain ^b^	15	nd	HS-SPME	GC-MS	Lilac aldehyde (I), lilac aldehyde (II), lilac aldehyde (IV), 3-methyl-1-butanol, lilac aldehyde (III)	[100]
Spain ^b^	10	nd	SDE	GC-MS	Furfural, methyl antranilate, phenylacetaldehyde, terpineal, 2-phenylethanol	[120]
Spain ^b^	4	nd	P&T	P&T-GC-MS	Heptane, 2,3-butanedione, dimethylsulfide, 2-methyl-3-buten-2-ol, octane	[101]
Spain ^b^	4	nd	SPME	GC-MS	Lilac aldehydes (isomers I-IV), *cis*-linalool oxide; phenylacetaldehyde; benzaldehyde; lilac alcohols (isomers (I-IV)	[79]
Spain ^b^	25	√	Trap with Tenax TA	GC-MS	Ethanol, 3-methyl-3-buten-1-ol, 2-methyl-1-butanol, 2-methyl-1-propanol, 2-methyl-3-buten-2 ol	[117]
Spain ^b^	>10	√	Trap with Tenax TA	GC-MS	Ethanol, acetone, pentane, benzenacetaldehyde, 2-methyl-1-propanol	[116]
Spain ^b^	8	√	HS-SPME	HS-SPME-GC-MS	Lilac aldehyde (isomer I), lilac aldehyde (isomer III), herboxide isomer II, dill ether, nonanal	[121]
Turkey ^b^	1	√	SPME	GC-MS	Lilac aldehyde, *cis*-6-nonen-1-ol, santene, methyl anthranilate	[87]

CHO: Country of honey origin. ^a^ *Citrus sinensis*. ^b^ *Citrus* spp. #: Number of samples studied. nd: no data. MPA: Melissopalynological analysis. √: MPA performed. VIP: Volatiles isolation procedure. VA: Volatiles analysis. Ref: References. HS-SPME: Headspace solid-phase microextraction. DHS: Dynamic headspace. P&T: Purge and trap concentrator. SDE: Simultaneous extraction and distillation. USE: Ultrasonic solvent extraction. GC: Gas chromatography. GC-MS: Gas chromatography-mass spectrometry.

**Table 14:** Main volatiles of pine honey and honeydew, with reference to the country of origin, number of samples, isolation and analysis procedures and five main volatile components. Unless otherwise specified, the honeybee type was *A. mellifera*.

CHO	#	MPA	VIP	VA	Dominant Volatile Compounds	Ref.
Greece ^a^	22	nd	P&T	GC-MS	1.4-Dichloro-benzene, nonanal, octane, decanal, α-isophorone	[122]
Greece ^b^	3	nd	HS-SPME	HS-SPME-GC-MS	Nonanal, 1-nonanol, furfural, nonanoic acid ethyl ester, decanal	[88]
Turkey ^a^	22	nd	P&T	GC-MS	Nonanal, octane, α-pinene, decanal, nonanol	[122]
Turkey ^b^	24	nd	SPME	GC-MS	Nonanal, nonanol, decanal, octanal, benzene acetaldehyde	[124]

CHO: Country of honey origin. ^a^ *Pinus* spp. honey. ^b^ *Pinus* spp. honeydew #: Number of samples studied. nd: no data. MPA: Melissopalynological analysis. VIP: Volatiles isolation procedure. VA: Volatiles analysis. Ref: References. HS-SPME: Headspace solid-phase microextraction. P&T: Purge and trap system. GC: Gas chromatography. GC-MS: Gas chromatography-mass spectrometry.

**Table 15:** Rape honey’s main volatiles, with reference to the country of origin, number of samples, isolation and analysis procedures and five main volatile components. Unless otherwise specified, the honeybee type was *A. mellifera*.

CHO	#	MPA	VIP	VA	Dominant Volatile Compounds	Ref.
Austria ^a^	1	nd	HS-SPME	GC-MS	2-Methylbutanoic acid, furfural, benzaldehyde, acetic acid, 2-phenylethanol	[41]
Belgium ^a^	5	√	DHS	GC and GC-MS	Octane, acetone, diacetyl, acetaldehyde, methyl formate	[76]
Denmark ^a^	1	√	Trap with Tenax TA	GC-MS	Acetoin, acetone, ethanol, nonanal, benzaldehyde	[52]
Estonia ^a^	3	√	SPME	GC-MS and GC-O	Dimethyl trisulphide, phenylacetaldehyde, hydrocinnamic acid, phenylacetic acid, *cis*-linalool oxide	[105]
France ^a^	1	√	DHS	GC and GC-MS	Octane, acetone, diacetyl, acetaldehyde, methyl formate	[76]
France ^a^	1	√	Trap with Tenax TA	GC-MS	Acetoin, acetone, ethanol, nonanal, benzaldehyde	[52]
Germany ^a^	2	√	Trap with Tenax TA	GC-MS	Acetoin, acetone, ethanol, nonanal, benzaldehyde	[52]
Germany ^a^	2	√	LLE	HRGC-MS and HRGC-O	Phenylacetic acid, 3-phenylpropanoic acid, 2-methylbutanoic acid, 3-methylbutanoic acid, benzyl alcohol	[125]
Lithuania ^a^	9	√	SPME	GC-MS	*p*-Cymenene, dimethyl sulphide, acetic formic anhydride, benzoic acid	[55]
Poland ^a^	8	nd	SPME	GC-MS and GC-O	Furfural, benzyl alcohol, 2-methylbutanol, 3-methylbutanol, *p*-cymen-8-ol	[72]
Poland ^a^	8	nd	HS-SPME	GC and GC-MS	Benzoic acid, benzyl alcohol, dimethyl disulphide, 1-nonanol, butyrolactone (dihydro-2(3h)-furanone)	[71]
Slovakia ^a^	7	nd	SPME	GC×GC-TOF-MS	Hexane, nonane, benzaldehyde, hotrienol, butan-2-one	[70]

CHO: Country of honey origin. ^a^ *Brassica napus*. #: Number of samples studied. nd: no data. MPA: Melissopalynological analysis. √: MPA performed. VIP: Volatiles isolation procedure. VA: Volatiles analysis. Ref: References. HS-SPME: Headspace solid-phase microextraction. LLE: Liquid–liquid extraction. DHS: Dynamic headspace. GC: Gas chromatography. GC-O: Gas chromatography-olfactometry. GC-MS: Gas chromatography-mass spectrometry. HR-GC-MS: High-resolution gas chromatography-mass spectrometry. HRGC-O: High-resolution gas chromatography-olfactometry. GC×GC-TOF-MS: Gas chromatography coupled to a time-of-flight mass spectrometer.

**Table 16:** Raspberry honey’s main volatiles, with reference to the country of origin, number of samples, isolation and analysis procedures and five main volatile components. Unless otherwise specified, the honeybee type was *A. mellifera*.

CHO	#	MPA	VIP	VA	Dominant Volatile Compounds	Ref.
Estonia ^a^	2	√	SPME	GC-MS and GC-O	1-Octen-3-one, butyric acid, phenylacetaldehyde, hydrocinnamic acid, hexyl hexanoate	[105]
Slovakia ^a^	2	nd	SPME	GC×GC-TOF-MS	Hexane, octane, nonane, decane, methyl ester of propanoic acid	[70]

CHO: Country of honey origin. ^a^ *Rubus idaeus*. #: Number of samples studied. nd: no data. MPA: Melissopalynological analysis. √: MPA performed. VIP: Volatiles isolation procedure. VA: Volatiles analysis. Ref: References. SPME: Solid-phase microextraction. GC-O: Gas chromatography-olfactometry. GC-MS: Gas chromatography-mass spectrometry. GC×GC-TOF-MS: Gas chromatography coupled to a time-of-flight mass spectrometer.

**Table 17:** Rhododendron honey’s main volatiles, with reference to the country of origin, number of samples, isolation and analysis procedures and five main volatile components. Unless otherwise specified, the honeybee type was *A. mellifera*.

CHO	#	MPA	VIP	VA	Dominant Volatile Compounds	Ref.
France ^a^	1	√	DHS	GC and GC-MS	Octane, acetaldehyde, acetone, ethyl formate, ethyl acetate	[76]
Hungary ^a^	1	√	DHS	GC and GC-MS	Octane, acetaldehyde, acetone, ethyl formate, ethyl acetate	[76]
Italy ^a^	3	√	HS-SPME	GC-MS	Ethanol, acetic acid, formic acid, furfural, hydroxyacetone	[77]
Spain ^a^	1	nd	SPME	GC-MS	3-Methyl-3-buten-1-ol, methyl-2-buten-1-ol, 3-methyl-1-butanol, *cis*-linalool oxide, benzaldehyde	[79]
Turkey ^a^	19	√	SPME	GC-MS	Benzene dicarboxylic acid, nonanal, 2-aminoacetophenone, isobutyl phthalate, *n*-decane	[87]
Turkey ^a^	14	√	SPME	GC-MS	1,2-Benzene dicarboxylic acid, stearic acid, tri-buthyl phosphate, benzophenone, ethyl phenyl acetate	[126]

CHO: Country of honey origin. ^a^ *Rhododendron* spp. #: Number of samples studied. nd: no data. MPA: Melissopalynological analysis. √: MPA performed. VIP: Volatiles isolation procedure. VA: Volatiles analysis. Ref: References. HS-SPME: Headspace solid-phase microextraction. DHS: Dynamic headspace. GC: Gas chromatography. GC-MS: Gas chromatography-mass spectrometry.

**Table 18:** Rosemary honey’s main volatiles, with reference to the country of origin, number of samples, isolation and analysis procedures and five main volatile components. Unless otherwise specified, the honeybee type was *A. mellifera*.

CHO	#	MPA	VIP	VA	Dominant Volatile Compounds	Ref.
France ^a^	4	√	DHS	GC and GC-MS	Acetaldehyde, octane, dimethylsulphide, acetone, ethyl formate	[76]
Portugal ^a^	1	√	Trap with Tenax TA	GC-MS	Acetone, 2-pentanone, benzaldehyde, 4-oxoisophorone, furfural	[52]
Spain ^a^	2	√	DHS	GC and GC-MS	Acetaldehyde, octane, dimethylsulphide, acetone, ethyl formate	[76]
Spain ^a^	1	√	Trap with Tenax TA	GC-MS	Acetone, 2-pentanone, benzaldehyde, 4-oxoisophorone, methyl-2-butenal	[52]
Spain ^b^	4	nd	SPME	GC-MS	Ethanol, acetic acid, furfural, 2,3-butanediol, 1-hydroxy-2-propanone	[99]
Spain ^b^	10	√	L-N	GC and GC-MS	Ethyl laurate, farnesol, thymol, 5-hydroxymethylfurfural, 3-phenylpropionate	[78]
Spain ^a^	1	nd	SDE, LLE, SPE	GC-MS	LLE: *meso*-2,3-Butenediol, hydroxymethylfurfural, *levo*-2,3-butenediol, acetic acid, benzoic acid SDE: Phenylacetaldehyde, ethyl oleate, hexadecanoic acid, tricosane, furfural SPE: Hydroxymethylfurfural, 2-phenylethanol, benzoic acid, methylfuroate, dibutyl 1,2-benzenedicarboxylate	[64]
Spain ^a^	35	nd	HS-SPME	GC-MS	3-Methyl-1-butanol, 3-methyl-3-buten-1-ol, benzaldehyde, 2,3-butanedione, benzene acetaldehyde	[100]
Spain ^b^	2	nd	P&T	P&T-GC-MS	Ethanol, heptane, 2-methyl-3-buten-2-ol, 2-methyl-1-propanol, octane	[101]
Spain ^a^	10	nd	SDE	GC-MS	Furfural, 3,4,5-trimethylphenol, 2-phenylethanol, benzyl alcohol, nonadecane	[97]
Spain ^b^	4	nd	SPME	GC-MS	2,6,6-Trimethyl-2,4-cycloheptadien-1-one (eucarvone), 3,5,5-trimethylcyclohex-2-ene-1-one (α-isophorone), 4-oxoisophorone, *cis*-linalool oxide, 2-methyl-1-butanol	[79]

CHO: Country of honey origin. ^a^ Rosemary. ^b^ *Rosmarinus officinalis*. #: Number of samples studied. nd: no data. MPA: Melissopalynological analysis. √: MPA performed. VIP: Volatiles isolation procedure. VA: Volatiles analysis. Ref: References. HS-SPME: Headspace solid-phase microextraction. DHS: Dynamic headspace. LLE: Liquid–liquid extraction. P&T: Purge and trap concentrator. SDE: Simultaneous extraction and distillation. SPE: Solid-phase extraction. L-N: Likens-Nickerson distillation extraction. GC: Gas chromatography. GC-MS: Gas chromatography-mass spectrometry.

**Table 19:** Strawberry tree honey’s main volatiles, with reference to the country of origin, number of samples, isolation and analysis procedures and five main volatile components. Unless otherwise specified, the honeybee type was *A. mellifera*.

CHO	#	MPA	VIP	VA	Dominant Volatile Compounds	Ref.
Greece ^a^	1	√	nd	GC-MS	3,5,5-Trimethyl-3-cyclohexen-1-one (β-isophorone), 3,5,5-trimethyl-2-cyclohexen-1-one (α-isophorone), 3,5,5-trimethylcyclohex-2-ene-1,4-dione (4-oxoisophorone)	[128]
Greece ^a^	4	nd	HS-SPME	HS-SPME-GC-MS	3,5,5-Trimethyl-2-cyclohexen-1-one (α-isophorone), 2,5-dimethyl-furan, nonanal, octane, 4-oxoisophorone	[88]
Italy ^a^	10	nd	DHS	GC-MS	α-Isophorone, β-isophorone, 4-oxoisophorone, 2,5-dimethylfuran, 2,3-butanedione	[127]
Spain ^a^	nd	nd	SPME	GC-MS	α-Isophorone, 2-hydroxy-3,5,5-trimethyl-2-cyclohexenone, 4-oxoisophorone, 2-furanmethanol, dimethyl sulphide	[104]

CHO: Country of honey origin. ^a^ *Arbutus unedo*. #: Number of samples studied. nd: no data. MPA: Melissopalynological analysis. √: MPA performed. VIP: Volatiles isolation procedure. VA: Volatiles analysis. Ref: References. HS-SPME: Headspace solid-phase microextraction. DHS: Dynamic headspace. GC: Gas chromatography. GC-MS: Gas chromatography-mass spectrometry.

**Table 20:** Sunflower honey’s main volatiles, with reference to the country of origin, number of samples, isolation and analysis procedures and five main volatile components. Unless otherwise specified, the honeybee type was *A. mellifera*.

CHO	#	MPA	VIP	VA	Dominant Volatile Compounds	Ref.
Czech Republic ^a^	10	√	Trap with Tenax TA	GC-MS	*n*-Decane, 2-butanone, ethanol, furfural, 3-methyl-3-buten-1-ol	[69]
France ^b^	4	√	DHS	GC and GC-MS	Acetaldehyde, octane, acetone, ethyl formate, diacetyl	[76]
France ^b^	2	√	Trap with Tenax TA	GC-MS	1-Butanol-3-methyl, 3-methyl-3-buten-1-ol, methyl-2-buten-1-ol, benzyl alcohol, benzaldehyde	[52]
Italy ^b^	2	√	Trap with Tenax TA	GC-MS	1-Butanol-3-methyl, 3-methyl-3-buten-1-ol, methyl-2-buten-1-ol, benzyl alcohol, benzaldehyde	[52]
Romania ^a^	10	√	Trap with Tenax TA	GC-MS	Furfural, *n*-decane, dimethyl sulfide, acetone, ethanol	[69]
Romania ^a^	8	√	SPE	GC-MS	*n*-Decane, 2-butanone, furfural, ethanol, methylsulfanylmethane	[111]
Slovakia ^a^	6	nd	SPME	GC×GC-TOF-MS	Hexane, octane, nonane, methyl ester of acetic acid, methyl ester of hexanoic acid	[70]
Spain ^a^	10	√	Trap with Tenax TA	GC-MS	*n*-Decane, furfural, 2-butanol, 2-butanone, dimethyl sulfide	[69]
Turkey ^b^	1	√	SPME	GC-MS	Benzene dicarboxylic acid, nonanal, damascenone, phenylacetaldehyde, α-α-dimethylphenyl acetate	[87]

CHO: Country of honey origin. ^a^ *Helianthus annuus*. ^b^ Sunflower. #: Number of samples studied. nd: no data. MPA: Melissopalynological analysis. √: MPA performed. VIP: Volatiles isolation procedure. VA: Volatiles analysis. Ref: References. HS-SPME: Headspace solid-phase microextraction. DHS: Dynamic headspace. SPE: Solid-phase extraction. GC: Gas chromatography. GC-MS: Gas chromatography-mass spectrometry. GC×GC-TOF-MS: Gas chromatography coupled to a time-of-flight mass spectrometer.

**Table 21:** Thyme honey’s main volatiles, with reference to the country of origin, number of samples, isolation and analysis procedures and five main volatile components. Unless otherwise specified, the honeybee type was *A. mellifera*.

CHO	#	MPA	VIP	VA	Dominant Volatile Compounds	Ref.
Greece ^a^	30	nd	USE	GC-MS	Hexadecanoic acid, tetracosane, veratric acid, 3-hydroxy-4-pheyl-3-buten-2-one, 1-phenyl-2,3-butanediol	[130]
Greece ^b^	42	√	HS-SPME	HS-SPME-GC-MS	Benzene acetaldehyde, benzaldehyde, 4-oxoisophorone, nonanoic acid ethyl ester, phenylethylalcohol	[132]
Greece ^b^	12	nd	HS-SPME	HS-SPME-GC-MS	Benzene acetaldehyde, benzaldehyde, benzeneethanol, benzeneacetonitrile, 2-furancarboxaldehyde (furfural)	[102]
Greece ^b^	31	√	HS-SPME	HS-SPME-GC-MS	Phenyleacetaldehyde, benzaldehyde, safranal, phenylacetonitrile, 2-furancarboxaldehyde (furfural)	[67]
Greece ^c^	4	nd	HS-SPME	HS-SPME-GC-MS	Nonanal, benzene acetaldehyde, nonanoic acid ethyl ester, decanal, benzeneethanol	[88]
Italy ^a^	2	√	SPME	GC and GC-MS	Ethenyl phenylacetate, α-hydroxybenzenepropanoic acid, 2-phenylethanol, phenylacetaldehyde, 2-(*p*-methoxyphenyl)ethanol	[75]
New Zealand ^c^	6	√	LLE	GC and GC-MS	1-(3-Oxo-*trans*-1-butenyl)-2,6,6-trimethylcyclohexane-*trans*, *cis*-1,2,4-triol, dimethyl butanedioate, methyl 3-hexenoate, 3’-aminoacetophenone, dimethyl *trans*-2-decenedioate	[133]
Palestine ^b^	nd	nd	HS-SPME	HS-SPME-GC-MS	1,3-diphenyl-2-propanone, (3-methylbutyl)-benzene, 3,4,5-trimethoxy benzaldehyde, 3,4-dimethoxy benzaldehyde, vanillin	[131]
Spain ^c^	4	nd	SPME	GC-MS	2,3-Butanediol, ethanol, acetic acid, 1-hydroxy-2-propanone, dimethyl sulfide	[99]
Spain ^c^	1	nd	P&T	P&T-GC-MS	Dimethylsulfide, 2-methyl-3-buten-2-ol, octane, acetonitrile, 2,3-butanedione	[101]
Spain ^a^	7	nd	SDE	GC-MS	Phenylacetaldehyde, furfural, 2-phenylethanol, octanoic acid, tetradecanoic acid	[97]
Spain ^c^	1	nd	SPME	GC-MS	Hotrienol, *cis*-linalool oxide, butanoic acid, phenylacetaldehyde, *p*-menth-1-en-9-al	[79]
Spain ^c^	30	√	P&T	GC-MS	Ethanol, heptanal, 2-methyl-1-propanol, 2-methyl-1-butanol, 2,3-butanedione	[49]
Turkey ^a^	7	nd	SPME	GC-MS	Dibenzylketone, 3-phenylhexane, *n*-octyl ether, phenylacetaldehyde, acetic acid	[129]

CHO: Country of honey origin. ^a^ Thyme. ^b^ *Thymus capitatus*. ^c^ *Thymus* spp. #: Number of samples studied. nd: no data. MPA: Melissopalynological analysis. √: MPA performed. VIP: Volatiles isolation procedure. VA: Volatiles analysis. Ref: References. HS-SPME: Headspace solid-phase microextraction. LLE: Liquid–liquid extraction. P&T: Purge and trap concentrator. SDE: Simultaneous distillation-extraction. USE: Ultrasound-assisted extraction. GC: Gas chromatography. GC-MS: Gas chromatography-mass spectrometry.
